# Monitoring phage-induced lysis of gram-negatives in real time using a fluorescent DNA dye

**DOI:** 10.1038/s41598-023-27734-w

**Published:** 2023-01-16

**Authors:** Julia E. Egido, Catherine Toner-Bartelds, Ana Rita Costa, Stan J. J. Brouns, Suzan H. M. Rooijakkers, Bart W. Bardoel, Pieter-Jan Haas

**Affiliations:** 1 Medical Microbiology, University Medical Center Utrecht, Utrecht University, Utrecht, The Netherlands; 2 Department of Bionanoscience, Delft University of Technology, Delft, The Netherlands; 3 Kavli Institute of Nanoscience, Delft, The Netherlands; 4 Fagenbank, Delft, The Netherlands

**Keywords:** Bacteriophages, Clinical microbiology

## Abstract

Bacteriophages (phages) are viruses that specifically attack bacteria. Their use as therapeutics, which constitutes a promising alternative to antibiotics, heavily relies on selecting effective lytic phages against the pathogen of interest. Current selection techniques are laborious and do not allow for direct visualization of phage infection dynamics. Here, we present a method that circumvents these limitations. It can be scaled for high-throughput and permits monitoring of the phage infection in real time via a fluorescence signal readout. This is achieved through the use of a membrane-impermeant nucleic acid dye that stains the DNA of damaged or lysed bacteria and new phage progeny. We have tested the method on *Pseudomonas aeruginosa* and *Klebsiella pneumoniae* and show that an increase in fluorescence reflects phage-mediated killing. This is confirmed by other techniques including spot tests, colony plating, flow cytometry and metabolic activity measurements. Furthermore, we illustrate how our method may be used to compare the activity of different phages and to screen the susceptibility of clinical isolates to phage. Altogether, we present a fast, reliable way of selecting phages against Gram-negative bacteria, which may be valuable in optimizing the process of selecting phages for therapeutic use.

Bacteriophage (phage) therapy has recently regained interest as an alternative to antibiotics, due to the increased prevalence of multidrug resistant bacteria and the limited availability of new antibacterial compounds^1,2^. This strategy is based on using phages, viral predators of bacteria, to treat bacterial infections in patients^3^. Phages are the most abundant biological entities on the planet, and they are immensely varied^4^. This diversity can constitute an advantage when searching for a phage to target a certain pathogen. On the other hand, phages have a narrow host range and often only infect a single species or even a specific strain. This means that therapeutic preparations must be tailored to specifically target the causative pathogen. Therefore, therapeutic phage preparations generally consist of a cocktail of different phages, with the aim of broadening the host range and overcoming any resistance that might arise^5,6^. Overall, selecting the optimal mix of phages for anti-bacterial therapy can be a challenging task. Hence, it is important to have methodologies that allow us to compare the activity of different phages.

Phages can follow different types of infection cycles. Some, referred to as temperate phages, can lysogenize the bacterial host, meaning that they are able to integrate their genome into the bacterial chromosome^7^. In contrast, phages used in therapy are often obligately lytic^8^. These phages first inject their genetic material into the host and then hijack the host’s cellular machinery to replicate and assemble into new viral particles. After enough progeny has been produced, the new phages will burst out of the host cell, causing it to lyse.

In phage therapy, an important step is the identification of phages that are able to infect the pathogen of interest. Traditionally, this is performed via a plaque assay^9^, in which phage suspensions are spotted onto a bacterial lawn^10^. Where the phages are causing bacterial growth inhibition or lysis, a clear area, named plaque, appears in the bacterial lawn. Another variation of this method, known as double-layer agar (DLA) assay, consists in mixing bacteria with phages in soft agar and overlaying that on a solid agar plate^11^. In both cases, the number of infective phages can be determined as plaque forming units by counting the single plaques that are obtained. These classical methods, while broadly accepted and inexpensive, cannot look at the dynamics of phage infection, and only provide results after overnight incubation. Furthermore, they are difficult to scale up for high-throughput screening, and they are not always precise or reproducible^12^.

In an effort to overcome these limitations, different ways to assess killing by bacteriophages have been described^13^. One of these is to measure the optical density of a bacterial culture and study how it changes over time as the phage infection progresses^14,15^. While this type of assay does allow for real-time monitoring of the infection, it is an indirect determination of bacterial damage since it only measures turbidity of the bacterial culture. Approaches based on bacterial respiration have also been explored^16,17^. Another assay that may be used is a one-step growth curve. In this technique, bacteria and phages are incubated together and aliquots of free phage, taken at different time-points, are plated on double-layer agar containing the host bacteria^18^. This assay is considered a golden standard when it comes to characterizing phages. It can be used to determine their latent period (time to generate new progeny), and the number of new phages produced per bacterium (burst size). However, this assay is very labor intensive, and, like the DLA assay, it is difficult to scale up. Therefore, it seems like complementing these assays with new high-throughput methods could aid the selection of phages for therapeutic use.

With this study, we aimed to develop a robust assay to monitor phage infections in real time in a multi-well plate setting. We use Sytox green, a membrane-impermeant nucleic acid dye that stains DNA of lysed bacteria and new phage progeny, producing a fluorescent signal as phage infection progresses. This tool has been extensively used to evaluate viability in bacteria^19^, but, to our knowledge, never to monitor killing by phages. Here, we show this application on *Pseudomonas aeruginosa* and *Klebsiella pneumoniae*, two ESKAPE pathogens against which phage therapy is particularly relevant^20,21^. We demonstrate that the developed assay correlates with standard assays, such as plaque assays and bacterial viability on plate, and propose a way of optimizing the method for phage screening in a clinical setting.

## Results

### Signal increase in fluorescent DNA dye assay reflects phage-mediated killing

**Figure 1:**
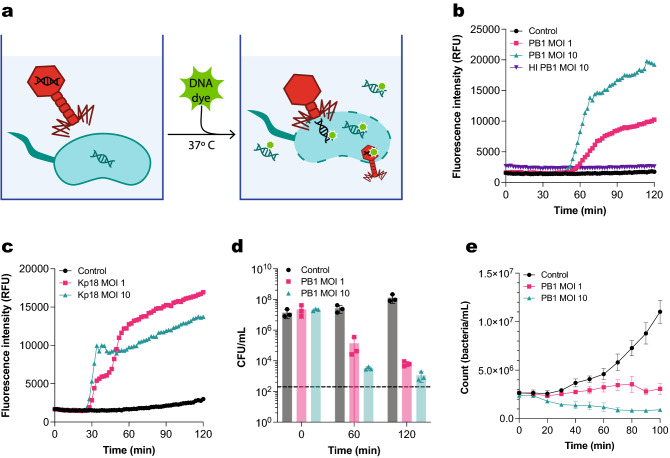
Fluorescent DNA dye assay detects phage-mediated killing. (**a**) Phage infection can be monitored in a 96-well plate setting using a membrane-impermeant DNA dye. The dye is added to a plate containing a phage-infected bacterial culture, which is then incubated at 37 °C. As the infection progresses, Sytox green will bind to phage DNA, DNA of damaged bacteria, and free bacterial DNA. The fluorescent signal of the DNA dye is measured over time. (**b**) Fluorescence intensity over time of *P. aeruginosa* infected with phage PB1 at an MOI of 1 and 10. Control are uninfected bacteria. Bacteria infected with heat-inactivated (HI) PB1 at an MOI of 10 are shown as a further control. (**c**) Fluorescence intensity over time of *K. pneumoniae* infected with phage Kp18 at an MOI of 1 and 10. Control are uninfected bacteria. (**d**) *P. aeruginosa* infected with phage PB1 at an MOI of 1 (pink) and 10 (green), or not infected (control, black) were plated at the initial time-point (0 min), after 60 min and after 120 min. Number of recovered colonies per plated volume is expressed as colony forming units per mL (cfu/mL). Black dotted line represents detection limit. (**e**) Bacterial concentration (count/mL) of a GFP-positive *P. aeruginosa* population in the first 100 min after infection with phage PB1 at an MOI of 1 or 10, as determined by means of flow cytometry. The concentration of an uninfected population is shown as control. (**b**, **c**) A representative graph of at least three independent experiments is shown. (**d**, **e**) Data represent mean ± SD of three independent experiments.

In order to measure phage infection in real time and in a high-throughput manner, we developed a fluorescence-based assay using the dye Sytox green. This dye cannot permeate through intact bacterial membranes, but it can enter both damaged bacteria and the capsid of non-enveloped bacteriophages (Fig. 1a)^19,22^. Thus, as a phage infection takes place, Sytox green will bind to free DNA, DNA of lysed or damaged bacteria, and phage DNA, emitting a strong fluorescent signal. The assay, hereafter referred to as fluorescent DNA dye assay, can be performed in a microplate reader.

To confirm the potential application of our fluorescent DNA dye assay for monitoring phage-induced damage, we looked at the infection of *P. aeruginosa* strain PAO1 by the strictly virulent myophage PB1. Phage PB1 was first identified by Bradley in 1966^23^ and it gives name to a very widely spread genus of phages infecting *P. aeruginosa*, Pbunavirus^24,25^. Some PB1-like viruses, like phage 14–1, have been included in phage therapy cocktails, making Pbunavirus an interesting genus of phages to assess in our assay^5^.

The assay was set up by mixing the host bacteria with phage PB1 and the DNA dye Sytox green (Fig. 1a). The phage preparation used here was pre-treated with DNase and RNase to reduce background staining. The fluorescence intensity of the dye was monitored over time to assess the progression of the infection process. We used bacteria incubated with heat-inactivated PB1 and bacteria alone as a control. When measuring over time a sigmoid curve was obtained for the conditions containing active phages (Fig. 1b, Figure S1). Both control conditions showed no increase in fluorescence, indicating that the signal obtained in the conditions with active phages is specific to phage-induced damage. For active PB1 at MOIs of both 10 and 1 we observe a latent period of approximately 60 min, corresponding to the time it takes for new phage progeny to be produced until the new virions cause host lysis. After this time, the fluorescence intensity signal starts steadily increasing until it reaches a plateau after 100 min. The height of this plateau was lower when using an MOI of 1. Although the dye Sytox green can bind to phage DNA, it was not seen to have any effect on the infectivity of phage PB1 (Figure S2). Similar results were obtained when performing the assay on a clinical strain of *K. pneumoniae* with podophage vB_KpP_FBKp18 (φKp18) of the *Autographiviridae* family (Fig. 1c, Figure S3, S4), indicating the applicability of the assay to other Gram-negatives. However, in this case, a second increase in fluorescent signal can be observed with an MOI of 1 after the first plateau is reached, which could correspond to a second cycle of phage infection.

Next, we assessed whether the increase in fluorescent signal reflects killing of bacteria. We incubated bacteria with phages under the same conditions as for the fluorescent DNA dye assay and evaluated their viability by colony forming units at different time points (Fig. 1d). We could detect that, for both MOIs tested, phages caused a reduction in viability already after 60 min, coinciding with the initial increase in fluorescence intensity (Fig. 1b). However, it should be noted that the assessment of colony formation could show confounding results in this case, as phage infection will be ongoing during the overnight incubation. As a further confirmation, we assessed the concentration of intact bacteria in an infected culture of *P. aeruginosa* (PAO1) in real time by means of flow cytometry (Fig. 1e). To do so, we inoculated a culture of bacteria expressing green fluorescent protein (GFP) with phage PB1 at different MOIs, after which we measured aliquots at different time-points. Here, we could observe that the concentration of bacteria infected with an MOI of 10 was reduced to half. In addition, up to 20% of the bacteria remaining showed membrane damage, as indicated by Sytox blue influx (Figure S5). By comparison, the control population increased steadily over the assessed period of time and remained impermeable to Sytox. These observations indicate that an increase in DNA dye fluorescence indeed reflects bacterial killing.

### Staining of bacterial DNA drives signal increase in fluorescent DNA dye assay

**Figure 2:**
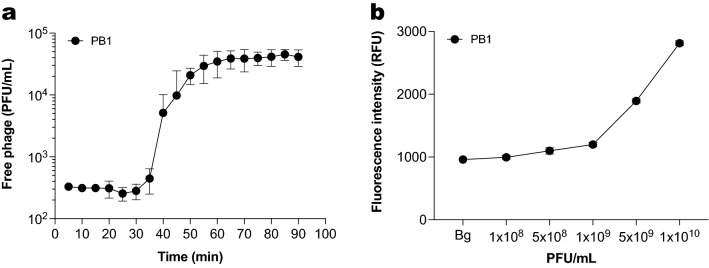
Staining of phage DNA is not the main driver of signal increase in the fluorescent DNA dye assay. (**a**) One-step growth curve of phage PB1. Concentration of phage is expressed as plaque-forming units per mL (pfu/mL). Data represent mean ± SD of three independent experiments. (**b**) Sytox green fluorescence intensity of phage PB1 at different concentrations. The background signal of the buffer is depicted as Bg. Data represent mean ± SD of three technical replicates.

As Sytox green can stain intact phages, the total signal might correspond not only to the DNA of damaged or lysed bacteria but also to newly produced phages. Therefore, we estimated how much the DNA of newly produced phages contributes to the fluorescent signal. We first determined the burst size of phage PB1, defined as the amount of new virions produced from each single infected bacterium^26^. This parameter can be obtained from a one-step growth curve, as described by Kropinski^18^. A one-step growth curve looks at the amount of free infective virions produced during the first cycle of phage replication after bacteria are infected. We obtained the one-step growth curve for PB1 (Fig. 2a) and based on this we calculated a burst size of 165 virions per bacterium (Figure S6). In the fluorescent DNA dye assay, the initial bacterial concentration is of 2 × 10^7^ bacteria/mL. Therefore, in an ideal situation where all bacteria lyse after the first infection cycle, the maximum concentration of phages obtained would be 3.3 × 10^9^ PFU/mL.

We then checked how much fluorescent signal would derive from staining phages in a similar concentration. Using Sytox green, we stained phage PB1 at several different concentrations between 10^8^ and 10^10^ PFU/mL and measured fluorescence intensity (Fig. 2b). Fluorescence intensity increased with phage concentration, indicating that Sytox green indeed stains encapsidated phage DNA. However, the phage-derived signal was at most three-fold higher than the background signal for all the concentrations assayed. In contrast, the maximum signal obtained in the fluorescent DNA dye assay after 2 h when bacteria are present is almost ten-fold higher than the background (Fig. 1b). This shows that the main contributor to the fluorescence signal in our assay is stained bacterial DNA (Figure S7), and not DNA of newly produced phages.

### Fluorescent DNA dye signal correlates with a loss of bacterial metabolic activity

To check if an increase in fluorescence signal corresponds to loss of bacterial viability in real time, we compared the results obtained in the fluorescent DNA dye assay with metabolic activity measurements. For this, we used *P. aeruginosa* with genomically integrated *lux* reporter genes^27^. These bacteria express the luciferase operon constitutively, and thus produce a luminescent signal if they are viable and metabolically active. The *lux* system, much like the fluorescent dye assay, allows for monitoring the state of the bacteria in real time by means of a plate reader. In addition, bacteria expressing the *lux* system can also be directly used in the fluorescent DNA dye assay. In this way, fluorescence and luminescence measurements can be obtained from the same well, which we used to further validate our fluorescent DNA dye assay.

**Figure 3:**
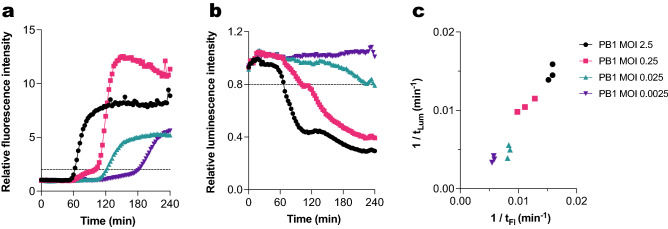
The fluorescent DNA dye signal correlates with a loss of bacterial metabolic activity. PAO1 expressing a luciferase reporter system was incubated with phage PB1 (different MOIs) at 37 °C in the presence of Sytox green. (**a**) Sytox green fluorescence intensity was measured using a fluorometer. Values were divided by the control signal (uninfected bacteria) to obtain relative fluorescence intensity. Black dotted line represents the threshold for phage-mediated damage (relative fluorescence = 2). (**b**) Luminescence intensity of samples was measured and divided by the signal of the control. Black dotted line represents threshold for phage-mediated damage (relative luminescence = 0.8). (**c**) Correlation between time to reach phage-mediated damage threshold as determined through fluorescent DNA dye staining and luminescence measurements for phage PB1 at different MOIs (Pearson’s r = 0.97, two-tailed *P* value < 0.0001). Data are represented as 1/t, where higher values indicate a faster infection. Data points represent individual results of three independent experiments. (**a**, **b**) A representative graph of at least three independent experiments is shown.

We incubated bacteria expressing the *lux* system with phage PB1 at different concentrations in the presence of Sytox green. Fluorescence measurements revealed a concentration-dependent effect, where higher MOIs caused an earlier signal increase (Fig. 3a, Figure S8). Luminescence measurements showed a similar MOI-dependent effect, where the highest MOI of 2.5 achieved a pronounced reduction of the luminescence signal, while the lower MOIs showed an intermediate effect (Fig. 3b, Figure S9). To more closely compare both signals, we established an arbitrary threshold for an increase in fluorescence and decrease in luminescence. When the signal crosses this threshold, we consider that we are detecting phage-induced damage. Based on this, we analyzed the time at which the phage-induced damage was detected by the fluorescent DNA dye assay (t_Fl_) and by luminescence measurements (t_Lum_). For each MOI, we plotted the inverse of t_Fl_ against the inverse of the corresponding t_Lum_, in such a way that higher values indicate a faster infection (Fig. 3c). This revealed a clear correlation between both types of measurements. We can thus conclude that a signal increase in the fluorescent DNA dye assay indicates loss of metabolic activity of the host bacteria.

### Bacterial metabolic activity correlates with fluorescent DNA dye assay for different *Pseudomonas* phages

**Figure 4:**
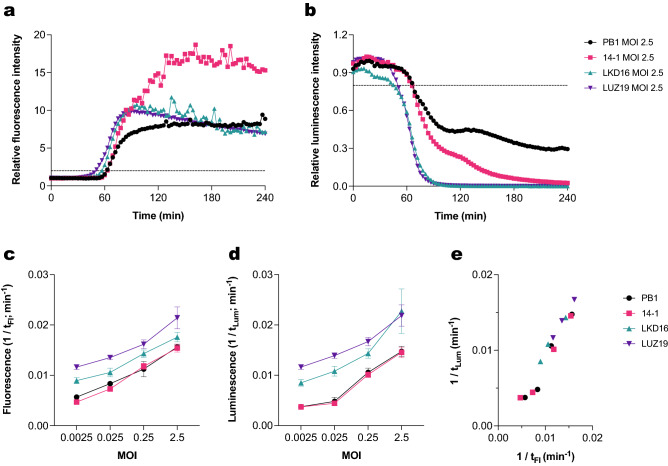
The fluorescent DNA dye assay correlates with bacterial metabolic activity for different *Pseudomonas* phages. PAO1 expressing a luciferase reporter system was incubated with phages PB1, 14–1, LKD16 and LUZ19 in a range of MOIs at 37 °C in the presence of Sytox green. (**a**) Sytox green fluorescence intensity was measured using a fluorometer. Values were divided by control signal to obtain relative fluorescence intensity. Black dotted line represents threshold for phage-mediated damage (relative fluorescence = 2). (**b**) Luminescence intensity of samples was measured and divided by signal of the control. Black dotted line represents threshold for phage-mediated damage (relative luminesence = 0.8). (**c**) Time of damage induction of the different phages as detected by the fluorescent DNA dye assay (t_Fl_). Data are plotted as 1/t_Fl_ and represent mean ± SD of three independent experiments. (**d**) Time of damage induction of the different phages as detected by luminescence measurements (t_Lum_). Data are plotted as 1/t_Lum_ and represent mean ± SD of three independent experiments. (**e**) Correlation between t_Fl_ and t_Lum_ for phages PB1, 14-1, LKD16 and LUZ19 at MOIs of 2.5, 0.25, 0.025 and 0.0025 (Pearson’s r = 0.97, two-tailed *P* value < 0.0001). Data represent mean of three independent experiments and are plotted as 1/t, where higher values indicate a faster infection. (**a**, **b**) A representative graph of at least three independent experiments is shown.

After confirming that the fluorescent DNA dye assay correlates with metabolic activity measurements in bacteria infected with PB1, we performed this analysis on a broader set of phages. We selected three additional well-described *Pseudomonas* lytic phages: 14–1^24^, LKD16^28^ and LUZ19^29^. When comparing all four phages using the fluorescent DNA dye assay, the podophages (LKD16 and LUZ19) caused a more rapid rise in fluorescence than the myophages (PB1 and 14–1) (Fig. 4a, Figure S10). Analysis of bacterial metabolic activity showed similar results (Fig. 4b, Figure S11). Phages 14–1, LKD16 and LUZ19 at an MOI of 2,5 caused metabolic activity to decrease to background levels, but the same was not observed for phage PB1. Lower MOIs of these phages caused slower and less pronounced changes in fluorescence and luminescence (Figures S12, S13).

We compared the time at which phage-induced damage was observed across all different conditions (t_Fl_, t_Lum_). For the fluorescent DNA dye assay, this revealed that the timing of damage induction depends on the MOI and on the type of phage. At higher MOIs, damage was detected faster for all the different phages (Fig. 4c). The same trend was observed when looking at loss of bacterial viability in terms of luminescence signal production (Fig. 4d). Therefore, we performed a correlation analysis between the time of phage-induced damage detected by both methods, including data from the four different phages at five different concentrations (Fig. 4e). Indeed, we found a significant correlation between these two parameters. This further confirms that an increase in fluorescence is related to a loss in bacterial metabolic activity. In addition, this analysis showcases the differences in kinetics between the four phages tested. Altogether, both methods appear to be suitable for assessing and comparing virulence of different phages.

### Fluorescent DNA dye assay facilitates susceptibility profiling of clinical isolates

Given the suitability of the fluorescent DNA dye assay for monitoring phage infections in bacteria, we analyzed if this method can screen clinical isolates for susceptibility to different phages. To standardize the assay and adapt it to a diagnostics laboratory workflow, we switched from using an overnight bacterial culture to using bacteria re-suspended directly from plate. This modification removes the need for overnight incubation and reduces the time needed to carry out the assay.

**Figure 5:**
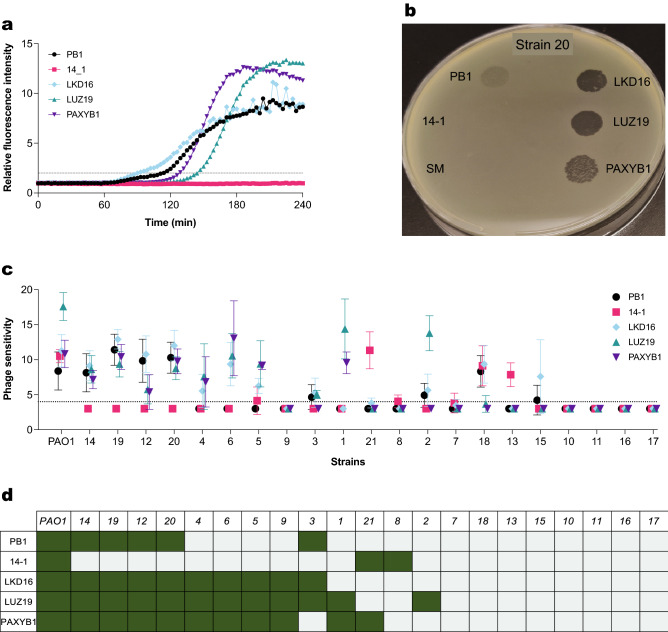
Phage susceptibility profiling of several *P. aeruginosa* clinical strains. (**a**) Strain 20 of the panel was incubated with phages PB1, 14-1, LKD16, LUZ19 or PAXYB1 at an MOI of 1 at 37 °C in the presence of Sytox green. Fluorescence intensity was measured using a fluorometer, and values were divided by control signal to obtain relative fluorescence intensity. Black dotted line represents threshold for phage-mediated damage (relative fluorescence = 2). A representative graph of at least three independent experiments is shown. (**b**) Strain 20 of the panel was inoculated into top agar and overlayed on a plate. Phages PB1, 14-1, LKD16, LUZ19 and PAXYB1 (10^6^ pfu/mL, 5 μL) and SM buffer (5 μL) were spotted to detect plaque formation after overnight incubation at 37 °C. (**c**) Summary of the susceptibility of *P. aeruginosa* strains to 5 different phages found with the fluorescent DNA dye assay. Phage sensitivity was defined as 1/time of damage * 1000 (min^−1^). A higher value reflects faster induction of phage-mediated damage. Black dotted line represents time detection limit in this assay (240 min). Data represent mean ± SD of three independent experiments. (**d**) Summary of the susceptibility to 5 different phages determined through plaque assays. Green indicates sensitivity to the phage (plaques were found), gray indicates no sensitivity (no plaques or barely detectable).

We then determined the susceptibility of a panel of 21 clinical *P. aeruginosa* isolates to different phages. These strains were obtained from patients suffering from chronic infections. We tested 5 phages of different taxa and targeting different receptors: 2 myophages targeting LPS (PB1 and 14–1)^24^ and 3 podophages targeting type 4 pili (LKD16, LUZ19 and PAXYB1)^28–30^. For each of the strains, we monitored phage-induced damage over the course of 4 h (Figs. 5a, S14) and analyzed the time of phage-induced damage (t_Fl_). Based on this parameter, we defined phage sensitivity as 1/(t_Fl_) × 1000 (Fig. 5c). We compared these results with a screening based on plaque assays (Figs. 5b, S15). Here, we spotted a high concentration of each of the phages on clinical strains to determine sensitivity in a coarse way. We scored phage sensitivity binarily depending on whether plaques were obtained (Fig. 5d).

For 95 out of the 110 phage-bacteria combinations tested (86%) both approaches yielded a similar result, where plaque formation corresponded to a positive fluorescent signal. No clear relationship was found between the timing of the fluorescent signal and the morphology or number of plaques. For instance, strain 20 seemed more quickly damaged by phage PB1 than by phage LUZ19 in the fluorescent DNA dye assay. However, this strain presented more turbid plaques for phage PB1 than for phage LUZ19. In addition, some discrepancies were found between both assays. Strains 2, 8, 13 and 15 showed an evident increase in fluorescent signal for several phages which did not seem infective in the plaque assays. Strikingly, the opposite effect was found for strain 9, which showed plaques for all 3 podophages and no increase in fluorescent signal. Another interesting observation is that phages with the same receptor were not necessarily able to infect the same strains, as seen for example for strain 1 (sensitive to LUZ19 and PAXYB1, but not LKD16) and strain 21 (sensitive to 14–1, but not to PB1). In conclusion, both assays could detect differences in the phage susceptibility profile of the clinical strains, producing matching but not completely overlapping results.

## Discussion

Finding suitable phages for specific clinical isolates can be a bottleneck in the process of administering phage therapy. Traditional methods, such as the DLA technique, are inefficient and offer very limited information regarding how the phage infection progresses. To circumvent this OD_600 nm_ measurements are often used. Although these can be collected in high-throughput and in real time, they do not directly measure damage and can therefore be difficult to interpret^13^. Other fast methods, such as the OmniLog™ system and surface plasmon resonance have been adapted to monitor phage infections, but these require specific equipment, making it more challenging to implement their use^31,32^. In sum, there is still room for improvement in methods for determining phage sensitivity, as there is currently no clear standard.

The present study tackles this problem by showing a way in which the DNA dye Sytox green can be used to monitor a lytic phage infection in real time. The application of DNA dyes as markers for cell death is widely accepted, both for eukaryotic and prokaryotic cells^33^. One example in which Sytox has been used in bacteria is to evaluate damage to Gram-negative outer membranes caused by the human innate immune system, especially the complement system^34^. This situation, however, is different to phage-mediated killing in that with the latter membrane damage is expected to occur from within, even leading to violent lysis^35^. Nonetheless, our results indicate that Sytox green can also have a valuable application for detecting phage-induced damage. A comparable finding was reported by Harhala et al., where Sytox green was used to effectively evaluate bacteriolytic activity of phage endolysins^36^, but this had not yet been shown for whole phage particles. Similarly, other nucleic acid dyes like SYBR gold and Syto 13 have been used to stain and detect phages and phage-infected bacteria, although these studies did not focus on assessing infection dynamics^37,38^.

We have shown that the fluorescent DNA dye assay presented here correlates with colony plating assays and measurements with bacteria expressing a *lux* reporter. Furthermore, we consider that our assay complements classical phage assessment methods in several aspects. First of all, it can follow the infection process from the moment bacteria and phages come in contact, although at low MOIs the resolution of the assay might not be sufficient to detect the first infection cycles. By monitoring phage-induced damage in time, infection dynamics of different phages can be compared. This information could potentially be useful for selecting phages for therapeutic cocktails, although further characterization of the phages would be necessary. Combining phages with different infection strategies may increase effectiveness^39^ and prevent development of phage resistance^40^. Other methods such as OD600 measurements also provide insights into the early stages of the infection process, but in some cases turbidity measurements are confounded by the optical density of bacterial debris and can be difficult to interpret. A one-step growth curve of the phage does provide more accurate information on the burst size, latent phase, and duration of a single infectious cycle, but this technique is not suited for testing different MOIs or different medium compositions. In addition, one-step growth curves are time-consuming, need to be repeated several times to obtain accurate representations, and can be performed for only a few phages at a time. Other plating-based methods where bacteria are evaluated after an overnight incubation, such as DLA, provide a more downstream readout than assays performed over time. Given that most phages have a latent period of less than an hour, it is crucial to observe the initial stages of phage-bacteria interactions. Furthermore, plating-based assays make it difficult to compare multiple conditions or phages simultaneously, and require considerable time and effort. Contrary to this, our method does not require labor intensive preparations and can be performed in medium to high-throughput, while ensuring that the assay conditions remain similar for all the phages being screened.

A limitation of the fluorescent DNA dye assay is that phage preparations need to be purified to a certain extent before using them in this assay; at least treated to remove nucleic acid remains. In addition, this system might not be suited to assessing infection by temperate phages. Phages that degrade host DNA, such as T4^41^, could also be difficult to assess. Nonetheless, the assay worked well for phage LUZ19, also known to partially degrade the host chromosome^29^, in the MOIs tested here. The fact that Sytox green can stain the DNA of non-enveloped phages is also a potential limitation^22^. However, our results show that the fluorescent signal that we obtain corresponds mainly to bacterial DNA. In addition, both membrane damage and release of new phage progeny are direct results of a productive infection. Therefore, the assay successfully monitors phage-induced damage regardless of whether the staining corresponds to phage or bacterial DNA. Still, it is difficult to interpret what the height of the fluorescent signal means in this assay, as a higher signal does not indicate more damage induction. For example, a population that is killed more slowly will continue dividing, thus accumulating more DNA and leading to a higher signal ultimately than one that is killed rapidly. This makes it challenging to estimate with this assay whether a whole population of bacteria has been killed. Nonetheless, the fluorescent DNA dye method gives clear information for phage therapy screening purposes, while more specialized assays can be used complementarily to determine the effect of a given phage on an entire bacterial population. To this effect, the *lux* system presented in the study can be particularly useful. Here, a total loss of luminescence is indicative of a total reduction in the bacterial population. While this system presents the disadvantage of requiring genetically modified bacteria, its use in combination with the fluorescence DNA dye assay is valuable when more precise characterization of a particular phage is needed.

We envision the fluorescent DNA dye assay as a tool to be used in clinical settings such as a diagnostic microbiology laboratory. Currently, there is no common accepted framework or pipeline to characterize and select phages for therapy, although some approaches have been suggested^42,43^. This, together with other factors such as stringent regulations, hampers the development of good phage therapy strategies^44^. Because of this, we believe it is worthwhile to optimize a high-throughput phage screening assay in a way that can fit into a diagnostic laboratory standard workflow. To this end, we showed that the DNA dye assay can be performed within 4 h of incubation. Using this method on clinical isolates revealed differences in their susceptibility profile to 5 different phages. The results obtained here were largely in agreement with plaque assays performed with the same phage-bacteria combinations. These were carried out by spotting a high number of PFU, and differences in sensitivity between strains were not quantified but rather expressed as a positive or negative result. In this analysis, plaque assays of isolates that were only partially sensitive to a certain phage were difficult to interpret, due to the appearance of ambiguous, turbid plaques, a phenomenon more often described in literature^45^. In addition, certain discrepancies were observed between both methods. In some cases, we could detect dye influx in bacteria incubated with a phage that did not form plaques on that strain, while in other cases the opposite effect was observed. This might be due to the high presence of phage defense systems in *P. aeruginosa* clinical isolates^46^. We hypothesize that certain abortive infection mechanisms might induce membrane permeabilization, potentially leading to dye influx but no plaque formation^47^. Conversely, high concentrations of phage may still form plaques on bacteria presenting different abortive infection mechanisms that would not cause a signal increase in the fluorescent dye assay^48^. In any case, further testing with a range of phage concentrations should be carried out in the cases where discrepancies are found to rule out possible false positive or false negative results. Another interesting observation is that strains sensitive to a given phage were not necessarily sensitive to another with the same receptor, further hinting at potential defense mechanisms at play. This highlights the need to focus on phage defense systems in addition to host range when selecting phages for therapeutic use^49^.

In summary, the fluorescent DNA dye assay that we present here represents a valid alternative to other existing methods and can offer additional information on how different phages behave. Moving forward, assays like this may be used in clinical settings to screen for suitable phages for personalized therapeutic approaches, although they would require significant validation before clinical use. The conditions used in this assay are easily adjustable, which would open up the possibility of including other factors in the equation, such as for instance patient serum or antibiotics. These prospects further encourage exploring new methods to develop more successful phage therapy strategies.

## Materials and methods

### Phages and strains

Stocks of *Pseudomonas* phages PB1, 14-1, LKD16 and LUZ19 were kindly provided by Dr Rob Lavigne (KU Leuven). *Pseudomonas* phage PAXYB1 was obtained from the Fagenbank (Delft, Netherlands). *Klebsiella* phage φKp18 was isolated from sewage water using *K. pneumoniae* L0549, a clinical isolate from UMCU, using an enrichment procedure as previously described^50^. Phage amplification was carried out by infecting the host strain (PAO1 for the *Pseudomonas* phages, L0549 for φKp18) overnight at 37 °C in Lysogeny Broth (LB). Bacterial debris was removed by centrifugation at 11,000 RCF for 40 min at 4 °C. Then, phages were incubated for 2 h on ice with a solution of 10% PEG-8000 and 0.5 M NaCl and precipitated by centrifugation at 11,000 RCF for 40 min at 4 °C. The preparation was mixed with chloroform, after which the aqueous phase was sterilized using a 0.2 μm filter and incubated with DNase and RNase (5 μg/mL each) for 30 min at room temperature. Final purity was achieved by filtration through a Zeba Spin desalting column (40 K MWCO, Thermo Fisher Scientific). PAO1 expressing GFP (PAO1 GFP +), encoded by plasmid pSMC21, was kindly provided by Dr Jeffrey Beekman. PAO1 expressing the *lux* system (PAO1-lux) was generated by transforming PAO1 with plasmid pTNS3 together with a plasmid encoding luxABCDE (pUC18-mini-Tn7T-Gm-lux)^51^. Clinical *P. aeruginosa* strains were obtained from the UMCU diagnostic microbiology laboratory strain collection and were isolated from patients with an invasive infection.

### Transmission electron microscopy

One ml of φKp18 phage lysate at a concentration higher than 10^9^ PFU/ml was sedimented by centrifugation at 21,000 × *g* for 1 h, washed and re-suspended in 1 ml of MilliQ water. Then, 3.5 µl of the phage were deposited and incubated for 1 min on TEM grids (Carbon Type-B 400 mesh, TED PELLA). The grids were washed three times with 40 µl of MilliQ water and stained with 3.5 µl of 2% (w/v) of uranyl acetate (pH 4.0) for 30 s. Grids were imaged using a JEM-1400 plus (JEOL) TEM.

### Bacteriophage host range

Ten-fold serial dilutions of the phages were spotted onto DLA plates of *K. pneumoniae* clinical strains L0506, L0549, K6310, L923, K6453, K6592, and K6500 (UMCU) and ATCC 11,296. The plates were incubated overnight at 37 °C and the phage plaques observed to distinguish productive infection and lysis from without^52^.

### Bacteriophage and bacteria genome sequencing

Bacterial DNA was extracted using the GeneJet Genomic DNA Purification kit (Thermo Fisher) and fragmented by Covaris 55 µL series Ultrasonicator. The DNA fragments were used to construct paired-end libraries with an insert size of 200–400 bp, and sequenced on the BGISEQ-500 (MGI, BGI-Shenzhen, China) platform. 1.4–2.0 Gb of sequencing data were generated for each sample with a sequencing depth of > 100x. Quality control of the raw data was performed using FastP^53^ and Soapnuke^54^ with default parameters, and the reads were trimmed and processed using Seqtk^55^. The filtered reads were assembled into the final genomes with SPAdes v3.13.0^56^. The capsular type of the *K. pneumoniae* strains was determined using Kaptive v0.7.3^57,58^ and the sequence type was determined using MLST 2.0^59^.

DNA of φKp18 was extracted using phenol–chloroform, and fragmented by Covaris 55 µL series Ultrasonicator. The DNA fragments were used to construct DNA nanoball (DNB)-based libraries by rolling circle replication, and sequenced using the BGI MGISEQ-2000 platform (BGI Shenzhen) with paired-end 100 nt strategy. This generated 4.6–19.2 Gb of sequencing data with a sequencing depth of > 10,000x.

### Bacteriophage genome annotation

Open reading frames (ORFs) of the φKp18 genome were predicted and automatically annotated using RAST sever v2.0^60^. Additional putative functions were assigned to ORFs by BlastP v.2.10.0^61^ and Hmmer v3.3.1^62^. Domains identified by Hmmer were included as ‘Notes’ in the annotation files. Schematics of phage genomes were built with the Linear Genomic Plot tool available at CTP Galaxy (https://cpt.tamu.edu/galaxy-pub).

### Microplate reader assays

Bacteria (PAO1 or PAO1-lux) were grown to mid-log phase (OD_600nm_ ~ 0.5) in LB, pelleted and resuspended to an OD_600nm_ of 1.0 (~ 8 × 10^8^ bacteria/mL) in RPMI 1640 (ThermoFisher) supplemented with 0.05% human serum albumin (HSA), then diluted 20-fold. Phages in SM buffer (100 mM NaCl, 8 mM MgSO_4_·7H_2_O, 50 mM Tris–Cl) were incubated with 10 μM Sytox green Nucleic Acid stain (ThermoFisher) at room temperature for up to 15 min. Equal volumes of bacteria and phages were then mixed obtaining a final concentration of ~ 2 × 10^7^ bacteria/mL bacteria and 5 μM Sytox green. Concentration of phages is dependent on the MOI, which is indicated for each experiment. Fluorescence and luminescence measurements were performed in a microplate reader (CLARIOstar, Labtech) at 37 °C. Assays where only fluorescence was measured were performed in a clear, flat-bottom 96-well plate (Corning), with the following settings: λ_excitation_ = 490 nm, bandwidth = 14 nm; λ_emission_ = 537 nm, bandwidth 30 nm; gain = 1300. Assays were both fluorescence and luminescence were measured were performed in white opaque 96-well plates (Corning), with the following settings: λ_excitation_ = 490 nm, bandwidth = 14 nm; λ_emission_ = 537 nm, bandwidth 30 nm; fluorescence gain = 1000; luminescence gain = 3600. For screening clinical isolates, the protocol was slightly modified. In this case, bacteria from a fresh blood agar plate were re-suspended in saline to a McFarland standard of ~ 2, and then diluted 1:20 in RPMI in a 96-well plate. Sytox green (final concentration: 5 μM) and phages (final concentration: 2 × 10^7^ PFU/mL) were added before measuring in a microplate reader as described above. The screening was performed three times; additional replicates are displayed in the supplementary materials (Figure S16).

### Determination of bacterial viability

Bacteria (PAO1) were grown to mid-log phase (OD_600nm_ ~ 0.5) in LB, pelleted and resuspended to an OD_600nm_ of 1.0 (~ 8 × 10^8^ bacteria/mL) in RPMI 1640 with 0.05% HSA. Bacteria and phages were mixed, adjusting the final concentration of bacteria to 2 × 10^7^ bacteria/mL. The phage concentration was determined according to the desired MOI. The mixture was incubated at 37 °C with shaking for 120 min. Samples were taken at t = 0 min, t = 60 min and t = 120 min. Serial dilutions were performed in RPMI 1640 with 0.05% HSA. 5 µL of each sample was plated on LB agar and incubated overnight at 37 °C. Colonies were counted, and the cfu/mL was calculated.

### Flow cytometry

Bacteria (PAO1 GFP +) were grown to mid-log phase (OD_600nm_ ~ 0.5) in LB, pelleted and resuspended to an OD_600nm_ of 1.0 (~ 8 × 10^8^ bacteria/mL) in RPMI 1640 with 0.05% HSA, then diluted 20-fold. Phages in SM buffer (concentration adjusted to the corresponding MOI) were mixed with bacteria in equal volumes. Sytox blue was added in a final concentration of 5 μM. The mixture was allowed to incubate at 37 °C with shaking. Samples were taken at the specified time-points and diluted tenfold in RPMI 1640 with 0.05% HSA before being analyzed. Flow cytometry was performed using the MACSQuant (Miltenyi biotech) by measuring the number of events in 10 uL of sample. Bacteria were gated based on GFP signal and forward scatters. The data were analyzed in FlowJo.

### One-step growth curve

One-step growth curves were conducted according to the method described by Kropinski^18^. Briefly, bacteria (PAO1) were grown to mid-log phase (OD_600nm_ ~ 0.5) in LB. Phage was prepared at 5 × 10^6^ pfu/mL in SM buffer. Phages were added 1:100 to the PAO1 culture and allowed to adsorb for 5 min at 37 °C with constant shaking. The mixture was diluted 1:100 in LB in flask A, which was then diluted 1:10 in LB in flask B, followed by a further 1:10 dilution in LB in flask C. 500 µL from flask A were added to 25 µL of ice-cold chloroform, vortexed and kept on ice to act as the adsorption control. 100 µL samples were taken at 5-min intervals from the appropriate flask (flask A in minutes 5–40, flask B in minutes 25–80, flask C in minutes 65–100), added to top agar along with 2 drops of an overnight culture of PAO1 and poured onto LB agar plates. After overnight incubation at 37 °C, plaques were enumerated, and pfu/mL was determined.

### Plaque assay

Bacteria were grown to mid-log phase (OD_600nm_ ~ 0.5) in LB. Top agar was prepared by mixing equal parts of LB broth and melted LB agar. After adjusting the temperature to 56 °C, 3 mL of top agar was mixed with 200 µL of bacterial culture and poured onto a pre-warmed LB agar plate to solidify. Phage suspensions in SM buffer (5 µL, 10^6^ PFU/mL) were spotted onto the solid top agar. SM buffer (5 µL) was spotted as a negative control. Plates were incubated overnight at 37 °C.

### Data analysis and statistical testing

Data visualization and statistical analyses were performed in GraphPad Prism 9 and are further specified in the figure legends.

## Supplementary Information

Supplementary Information.

## Data Availability

The datasets generated and analyzed during this study are presented in the supplementary materials. Raw data and assembled genomes of the *K. pneumonia* clinical strains and phage φKp18 are available from Genbank, Bioproject PRJNA745534.
