# Supplementary material for: Monitoring phage-induced lysis of gram-negatives in real time using a fluorescent DNA dye
Source: Sci Rep. 2023 Jan 16;13:856. doi: 10.1038/s41598-023-27734-w (PMC9842612; doi:10.1038/s41598-023-27734-w)
Supplement: Supplementary file 1 — Supplementary Information. [file 41598_2023_27734_MOESM1_ESM.pdf]

Supplementary materials

Monitoring phage-induced lysis of Gram-negatives in real time using a fluorescent DNA dye

Julia E. Egido[1], Catherine Toner-Bartelds[1], Ana Rita Costa[2,3,4], Stan J. J. Brouns[2,3,4], Suzan H. M. Rooijakkers[1], Bart W. Bardoel[1], Pieter-Jan Haas[1*]

[1]*Medical Microbiology, University Medical Center Utrecht, Utrecht University, Utrecht, Netherlands*
[2]*Department of Bionanoscience, Delft University of Technology, Delft, Netherlands*
[3]*Kavli Institute of Nanoscience, Delft, Netherlands*
[4]*Fagenbank, Delft, Netherlands*

*Corresponding author: p.j.a.haas@umcutrecht.nl

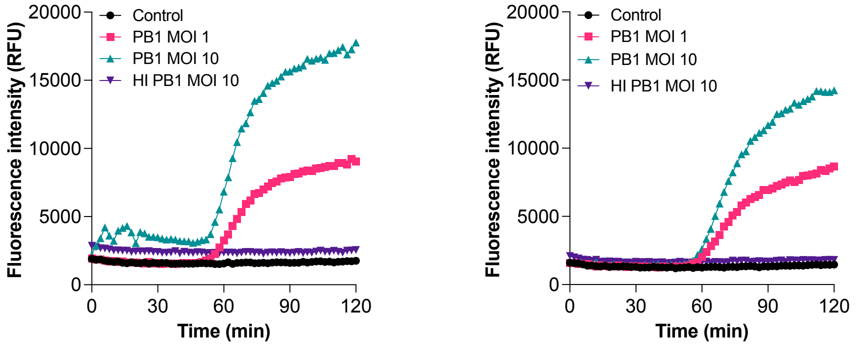

Figure S1. Fluorescence intensity over time of *P. aeruginosa* infected with phage PB1 at an MOI of 1 and 10. Control are uninfected bacteria. Bacteria infected with heat-inactivated (HI) PB1 at an MOI of 10 are shown as a further control. Two additional replicates to figure 1b are shown.

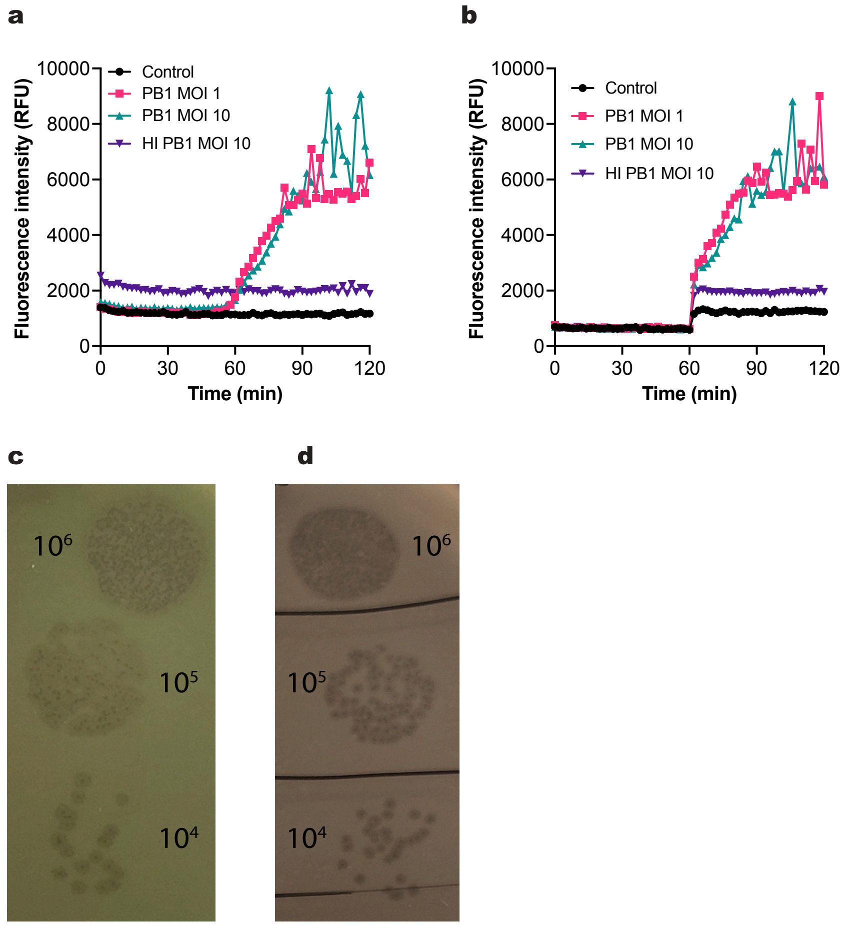

Figure S2. Infectivity of phage PB1 is not affected by Sytox green. a,b) Fluorescence intensity over time of *P. aeruginosa* infected with phage PB1 at an MOI of 1 and 10. Control are uninfected bacteria. Bacteria infected with heat-inactivated (HI) PB1 at an MOI of 10 are shown as a further control. Sytox green (5 μM) was added prior to measuring **(a)** or after 60 minutes **(b)**. **c,d)** Spot tests performed with PB1 incubated for 2 hours in absence **(c)** or presence **(d)** of Sytox green (5 μM). Estimated phage concentrations in pfu/mL are indicated.

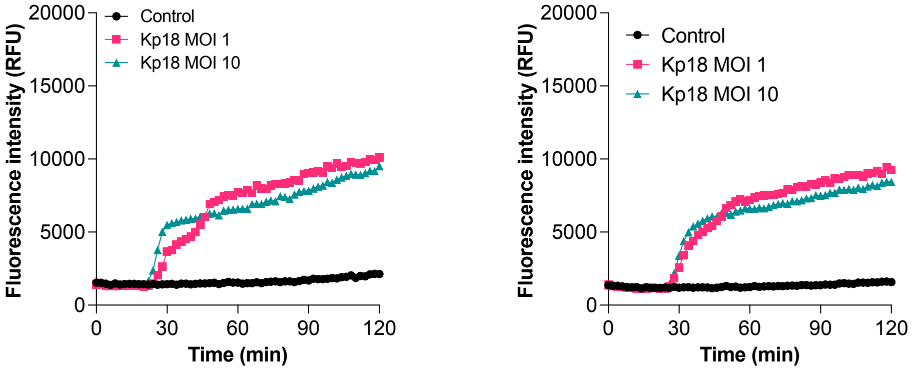

Figure S3. Fluorescence intensity over time of *K. pneumoniae* infected with phage Kp18 at an MOI of 1 and 10. Control are uninfected bacteria. Two additional replicates to figure 1c are shown.

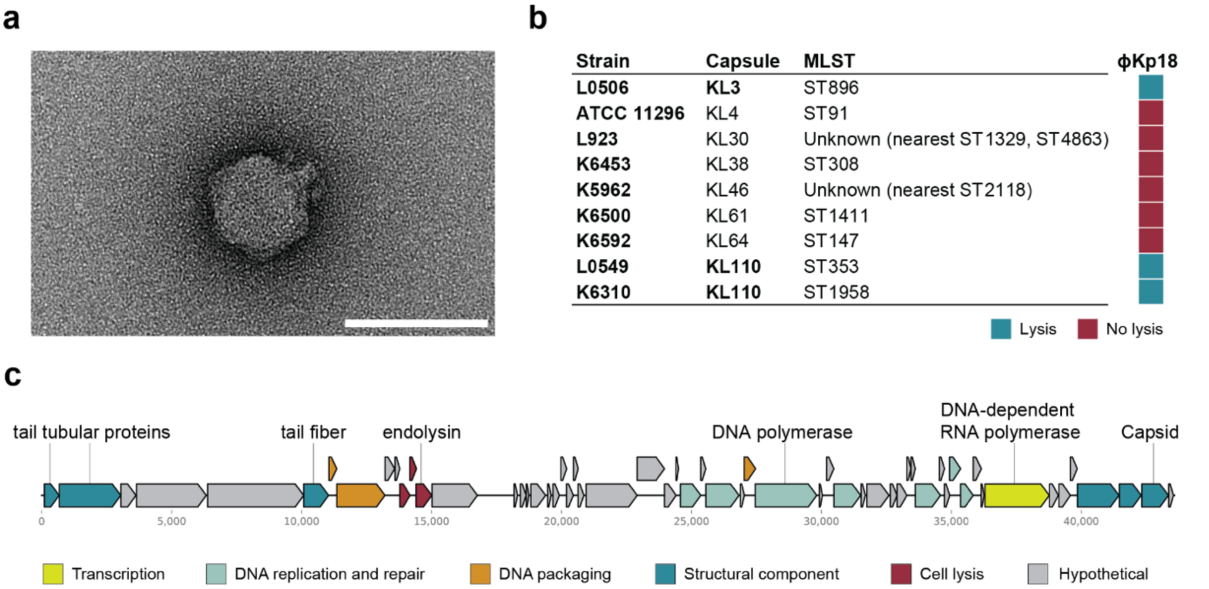

Figure S4. Characteristics of *Klebsiella* phage vB_KpP_FBKp18. a) Transmission electron microscopy image of ϕKp18 negatively stained with 2% uranyl acetate. Scale bar, 80 nm. Micrograph taken at 200,000x magnification. **b)** Infectivity of phage ϕKp18 against *K. pneumoniae* strains of different capsular (KL) and multiple sequence locus (MLST) types. **c)** Linear genome map of ϕKp18. ORFs are colored according to predicted function as shown in the key.

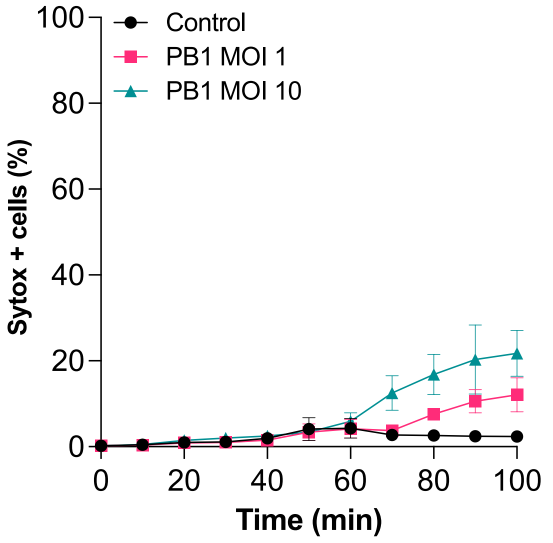

Figure S5. Part of the bacterial population can become permeable to the dye Sytox before lysing as a result of phage infection. Percentage of Sytox blue-positive cells of a GFP-positive *P. aeruginosa* population in the first 100 minutes after infection with phage PB1 at an MOI of 1 or 10, as determined by means of flow cytometry. An uninfected population is shown as control. Data represent mean ± SD of three independent experiments.

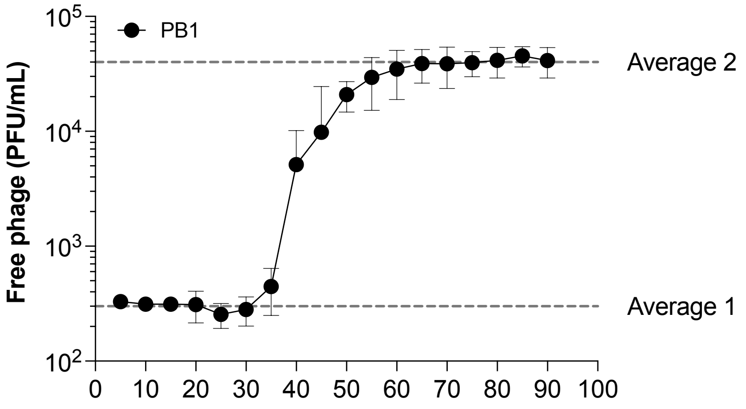

Average 1 = 300 PFU/mL

Average 2 = 40017 PFU/mL

Adsorption control (average) = 58 PFU/mL

Burst size = Average 2 / (Average 1 - Adsorption control) = 165

Figure S6. One-step growth curve of phage PB1 and calculation of its burst size, as described by Kropinski (2018)[14].

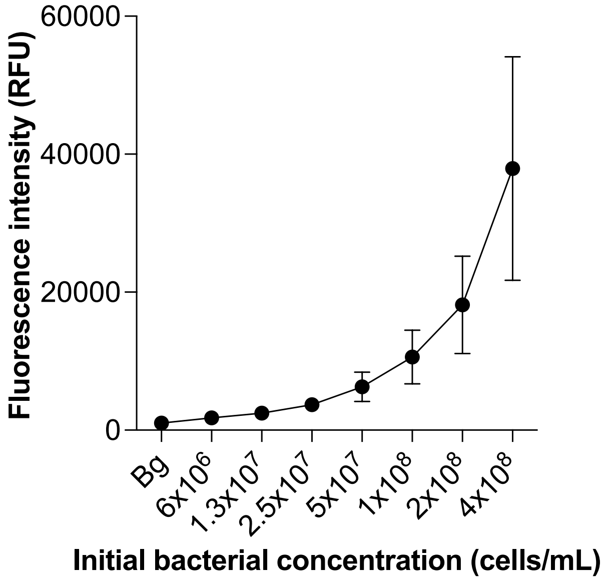

Figure S7. Fluorescence intensity of increasing concentrations of bacterial DNA stained with 5 μM Sytox green is not limited by the dye concentration. Bacteria (PAO1) in medium (RPMI 1640 with 0.05% HAS) at different concentrations were vortexed together with an equal amount of chloroform, then centrifuged shortly at high speed. The aqueous phase containing the bacterial DNA was mixed in a microplate with an equal volume of SM buffer to a final volume of 100 μL per well. Sytox green was added in a final concentration of 5 μM. Fluorescence intensity was measured in a microplate reader (CLARIOstar, Labtech) with the following settings: $\lambda_{excitation}$ = 490 nm, bandwidth = 14 nm; $\lambda_{emission}$ = 537 nm, bandwidth 30 nm; gain = 1300. Data represent mean ± SD of three biological replicates.

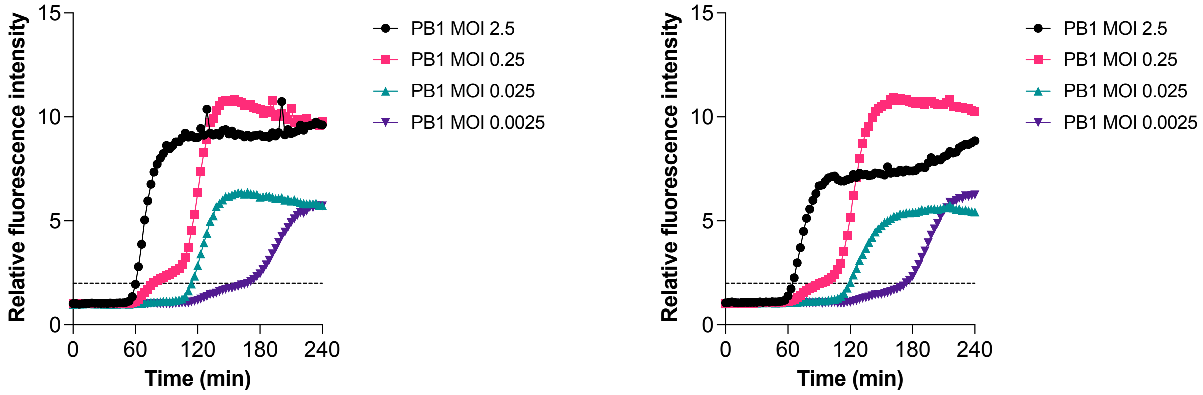

Figure S8. Relative fluorescence intensity over time of PAO1 expressing a luciferase reporter system incubated with phage PB1 (different MOIs) at 37ºC in presence of Sytox green. Sytox green fluorescence intensity was measured using a fluorometer. Values were divided by the control signal (uninfected bacteria) to obtain relative fluorescence intensity. Black dotted line represents the threshold for phage-mediated damage (relative fluorescence = 2). Two additional replicates to figure 3a are shown.

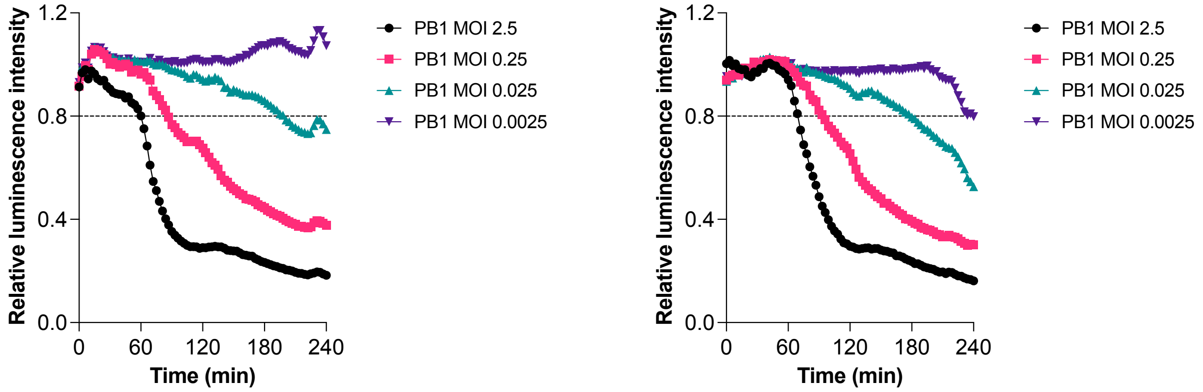

Figure S9. Relative luminescence intensity over time of PAO1 expressing a luciferase reporter system incubated with phage PB1 (different MOIs) at 37ºC in presence of Sytox green. Luminescence intensity values were divided by the control signal (uninfected bacteria) to obtain relative fluorescence intensity. Black dotted line represents the threshold for phage-mediated damage (relative fluorescence = 2). Two additional replicates to figure 3b are shown.

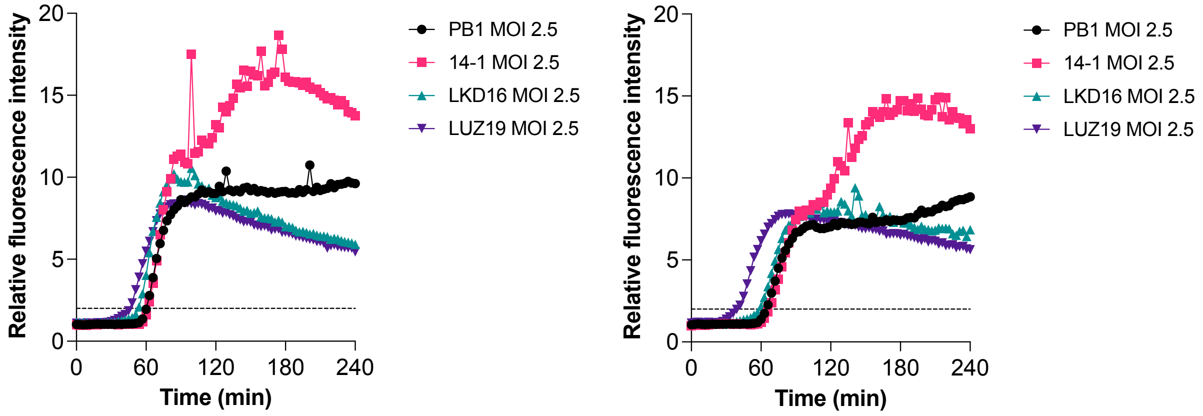

Figure S10. Relative fluorescence intensity over time of PAO1 expressing a luciferase reporter system incubated with phages (MOI = 2.5) at 37°C in presence of Sytox green. Sytox green fluorescence intensity was measured using a fluorometer. Values were divided by the control signal (uninfected bacteria) to obtain relative fluorescence intensity. Black dotted line represents the threshold for phage-mediated damage (relative fluorescence = 2). Two additional replicates to figure 4a are shown.

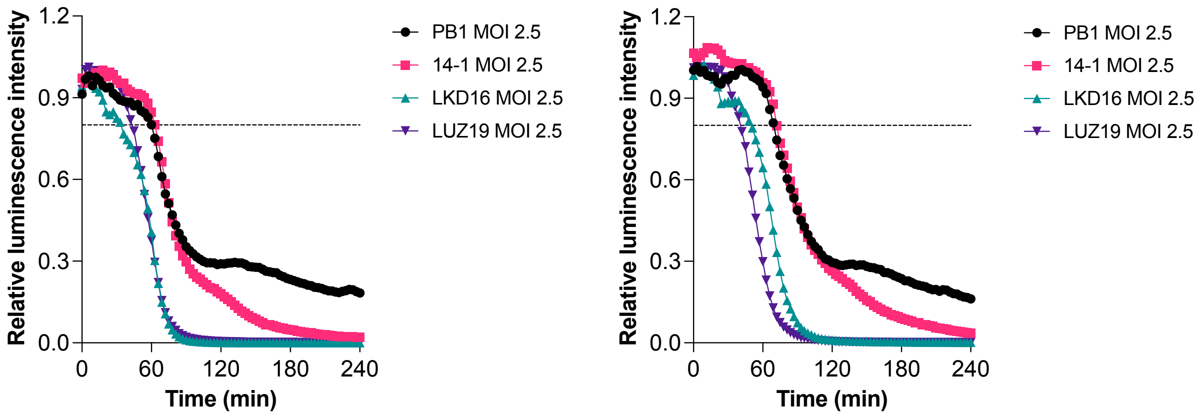

Figure S11. Relative luminescence intensity over time of PAO1 expressing a luciferase reporter system incubated with phages (MOI = 2.5) at 37°C in presence of Sytox green. Luminescence intensity values were divided by the control signal (uninfected bacteria) to obtain relative fluorescence intensity. Black dotted line represents the threshold for phage-mediated damage (relative fluorescence = 2). Two additional replicates to figure 4b are shown.

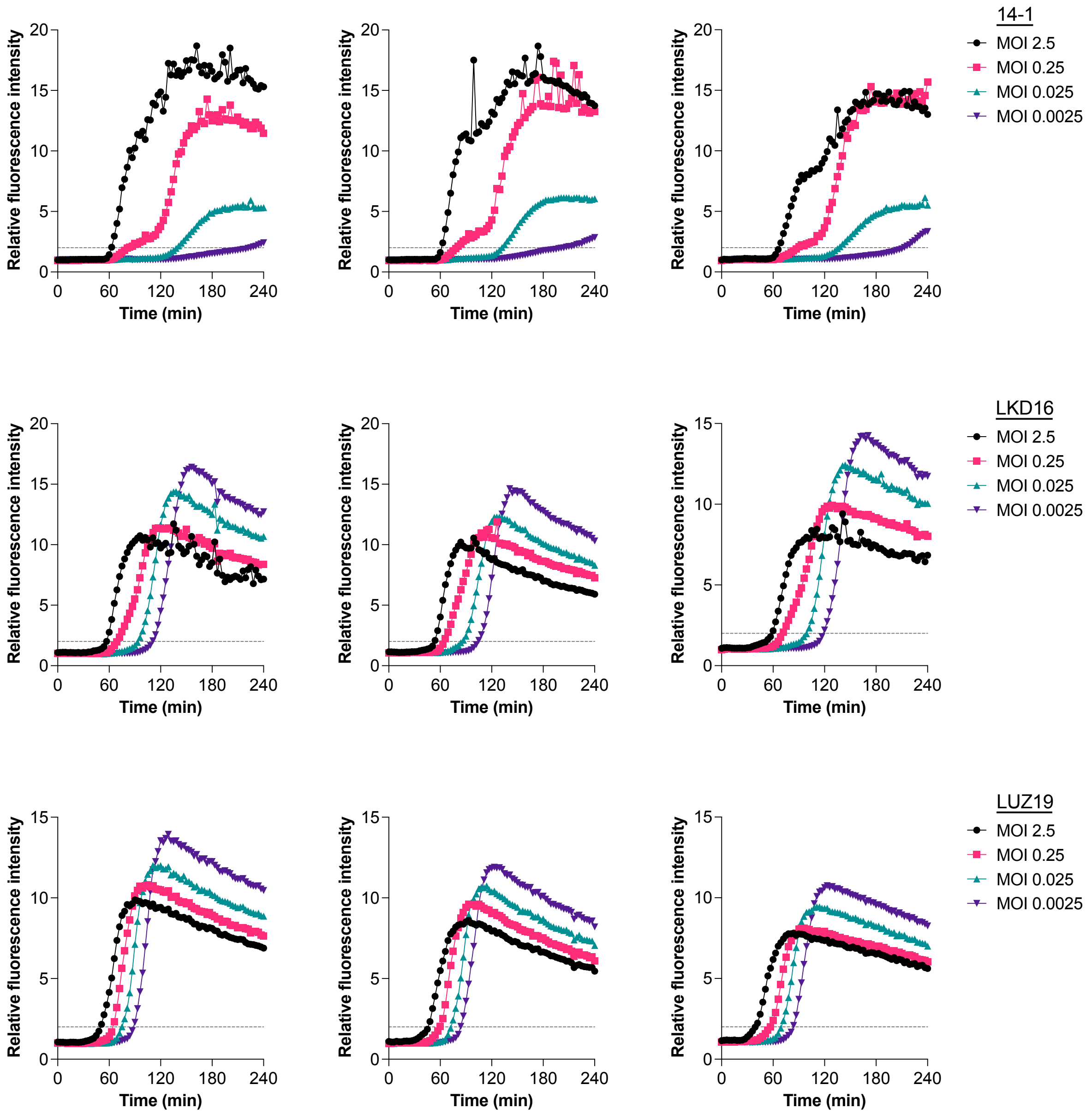

Figure S12. Relative fluorescence intensity over time of PAO1 expressing a luciferase reporter system incubated with phages (different MOIs) at 37°C in presence of Sytox green. Sytox green fluorescence intensity for phages 14-1, LKD16 and LUZ19 was measured using a fluorometer. Values were divided by control signal to obtain relative fluorescence intensity. Black dotted line represents threshold for phage-mediated damage (relative fluorescence = 2). Three independent experiments are shown.

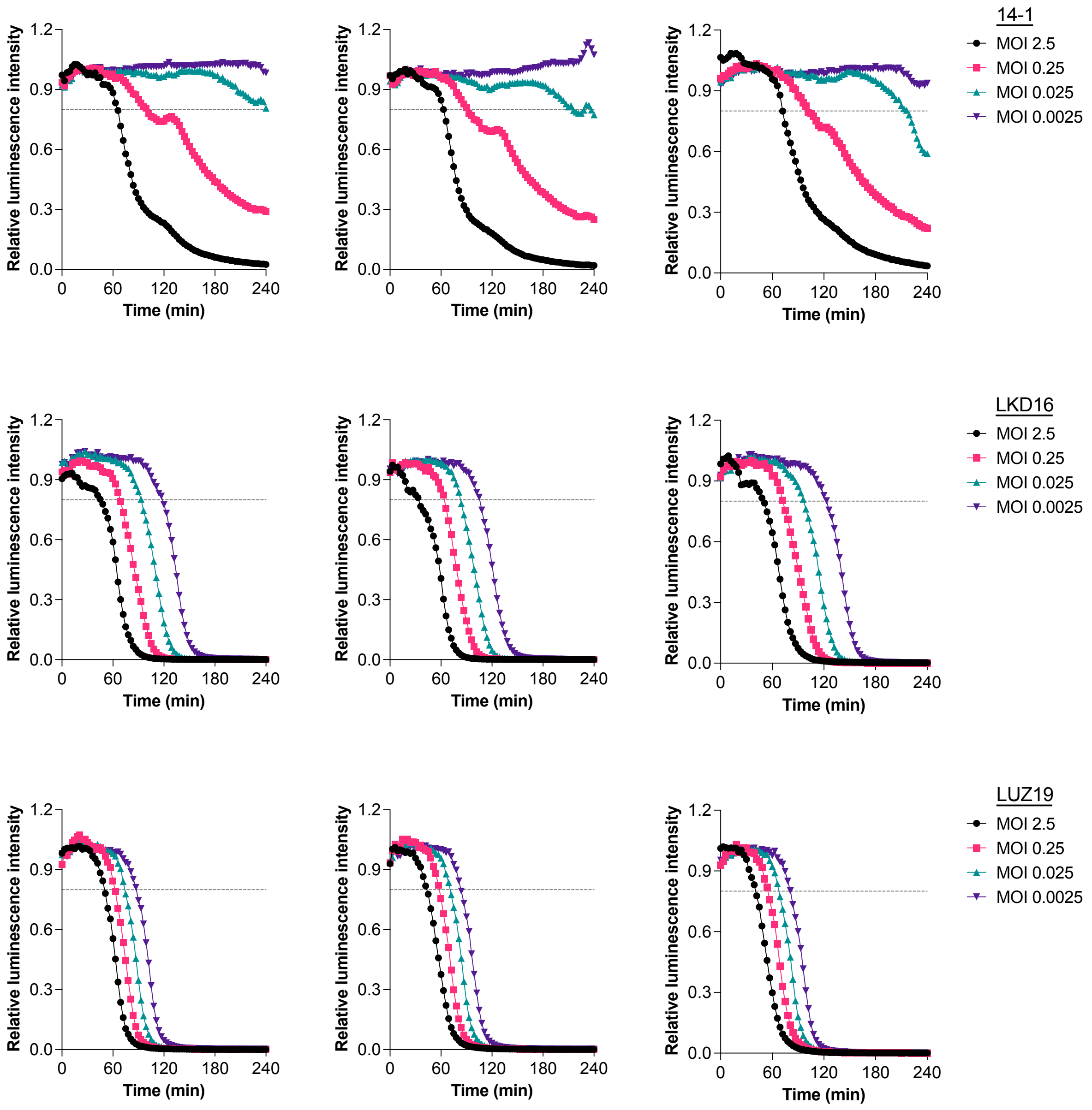

Figure S13. Relative luminescence intensity over time of PAO1 expressing a luciferase reporter system incubated with phages (different MOIs) at 37°C in presence of Sytox green. Luminescence intensity for phages 14-1, LKD16 and LUZ19 was measured. Values were divided by control signal to obtain relative luminescence intensity. Black dotted line represents threshold for phage-mediated damage (relative luminescence = 0.8). Three independent experiments are shown.

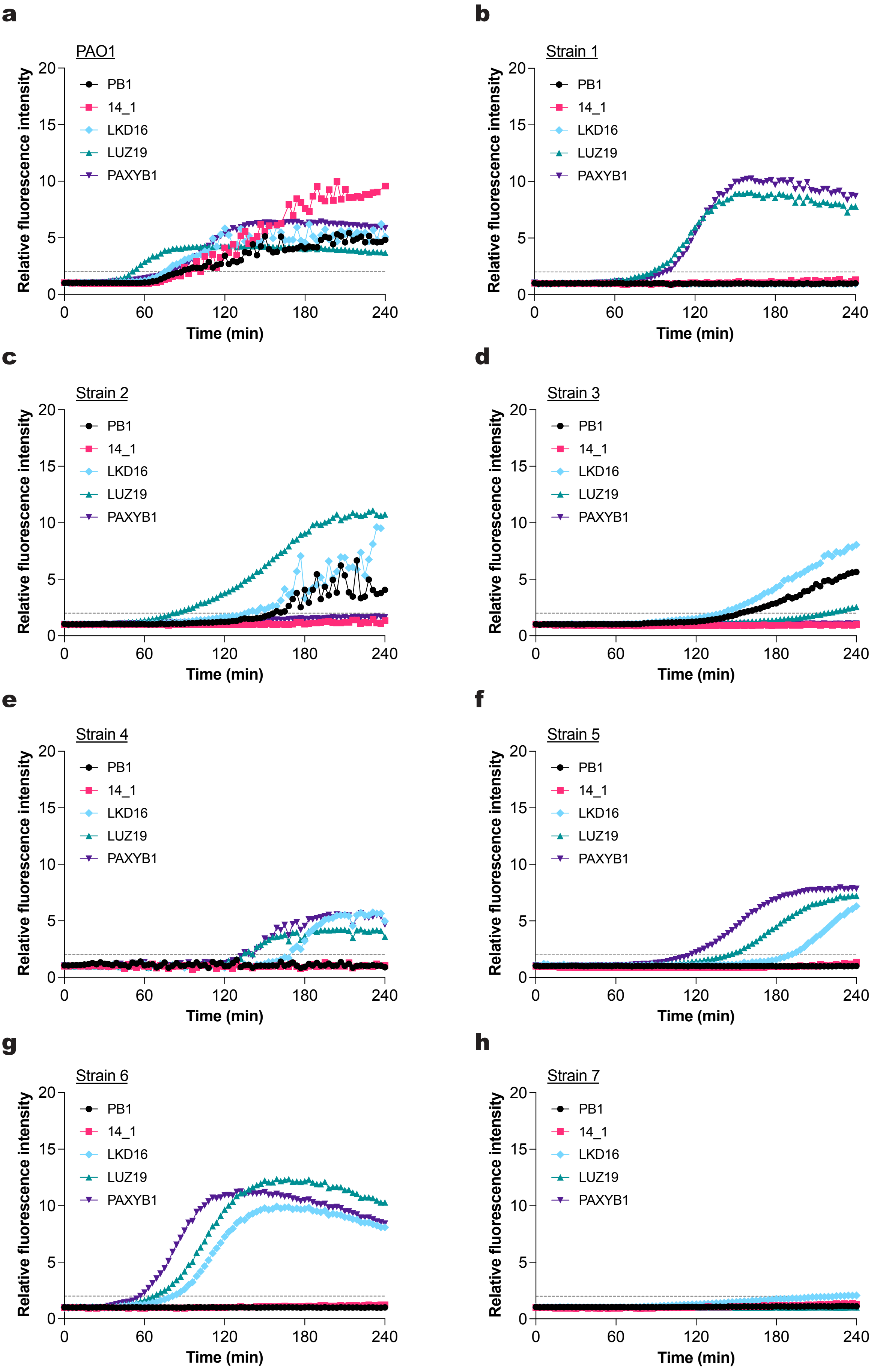
a
PAO1
PB1
14_1
LKD16
LUZ19
PAXYB1
Relative fluorescence intensity
Time (min)
b
Strain 1
PB1
14_1
LKD16
LUZ19
PAXYB1
Relative fluorescence intensity
Time (min)
c
Strain 2
PB1
14_1
LKD16
LUZ19
PAXYB1
Relative fluorescence intensity
Time (min)
d
Strain 3
PB1
14_1
LKD16
LUZ19
PAXYB1
Relative fluorescence intensity
Time (min)
e
Strain 4
PB1
14_1
LKD16
LUZ19
PAXYB1
Relative fluorescence intensity
Time (min)
f
Strain 5
PB1
14_1
LKD16
LUZ19
PAXYB1
Relative fluorescence intensity
Time (min)
g
Strain 6
PB1
14_1
LKD16
LUZ19
PAXYB1
Relative fluorescence intensity
Time (min)
h
Strain 7
PB1
14_1
LKD16
LUZ19
PAXYB1
Relative fluorescence intensity
Time (min)
0
5
10
15
20
0
60
120
180
240

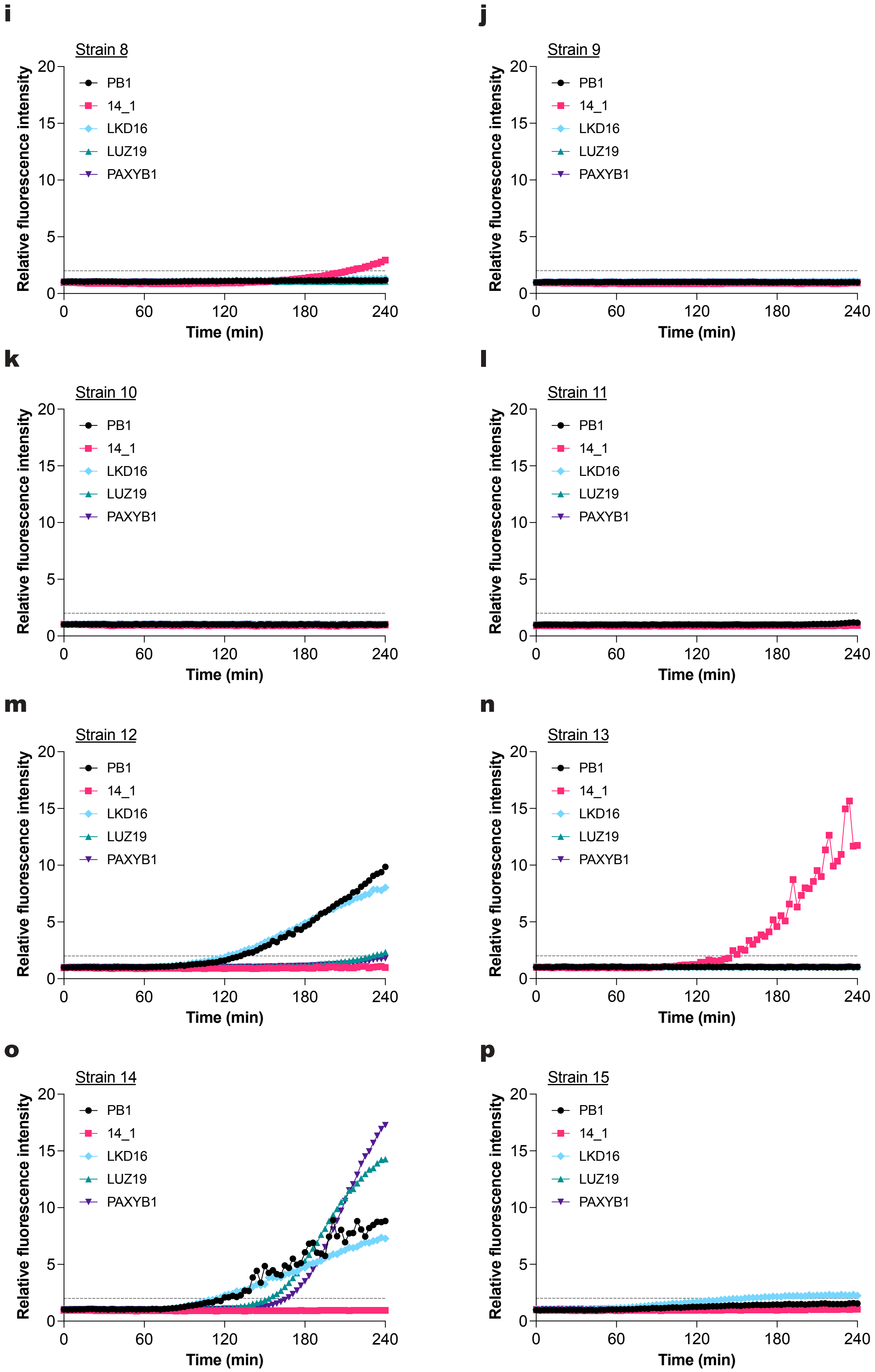
i
Strain 8
j
Strain 9
k
Strain 10
l
Strain 11
m
Strain 12
n
Strain 13
o
Strain 14
p
Strain 15
PB1
14_1
LKD16
LUZ19
PAXYB1
Relative fluorescence intensity
0
5
10
15
20
Time (min)
0
60
120
180
240

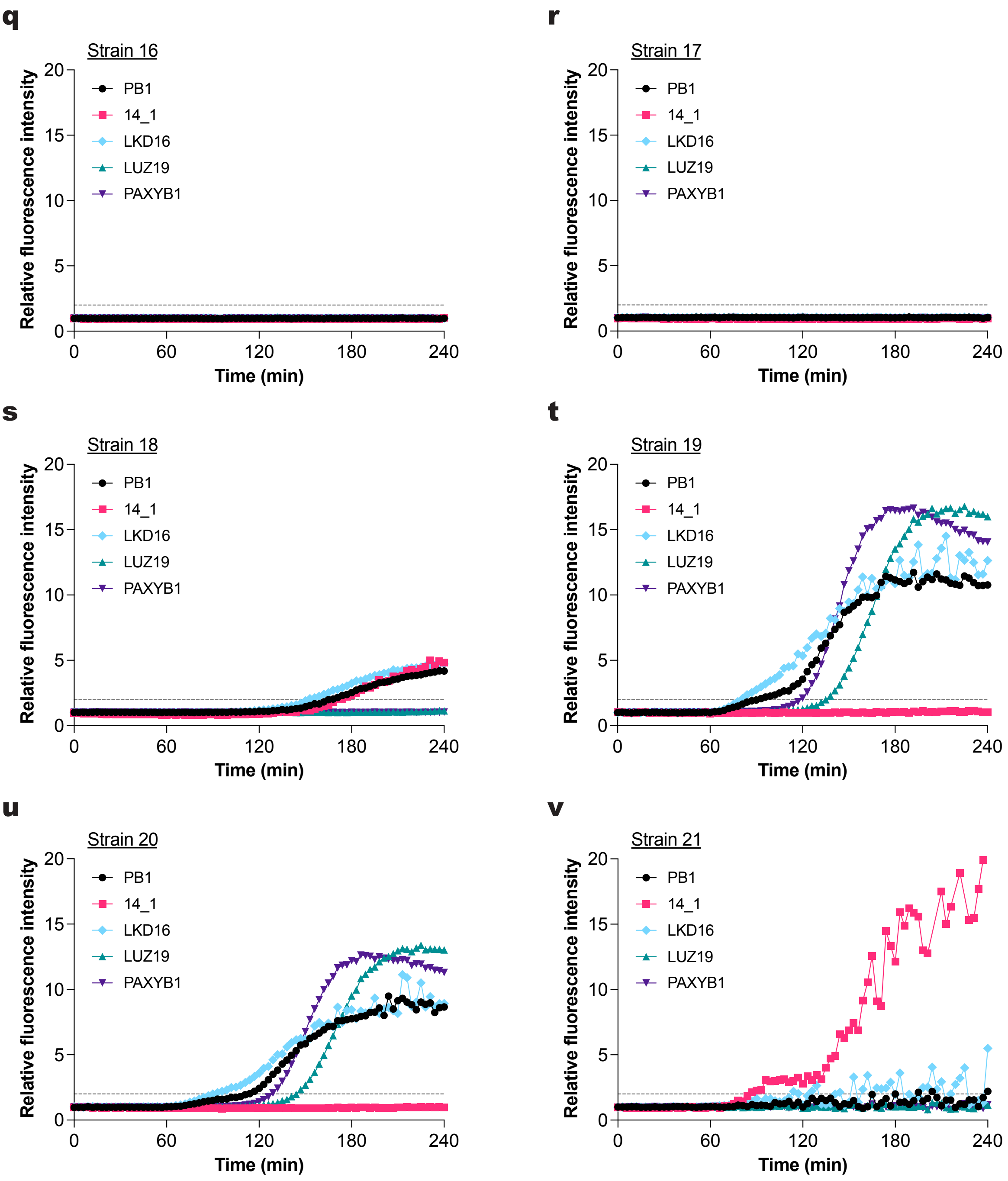

Figure S14: Phage susceptibility profiling of several *P. aeruginosa* clinical strains obtained from patients at the UMC Utrecht, as determined by fluorescent DNA dye assay. a-v) Strains were incubated with phages PB1, 14-1, LKD16, LUZ19 or PAXYB1 at an MOI of 1 at 37°C in the presence of Sytox green. Fluorescence intensity was measured using a fluorometer, and values were divided by control signal to obtain relative fluorescence intensity. Black dotted line represents threshold for phage-mediated damage (relative fluorescence = 2). A representative graph of at least three independent experiments is shown.

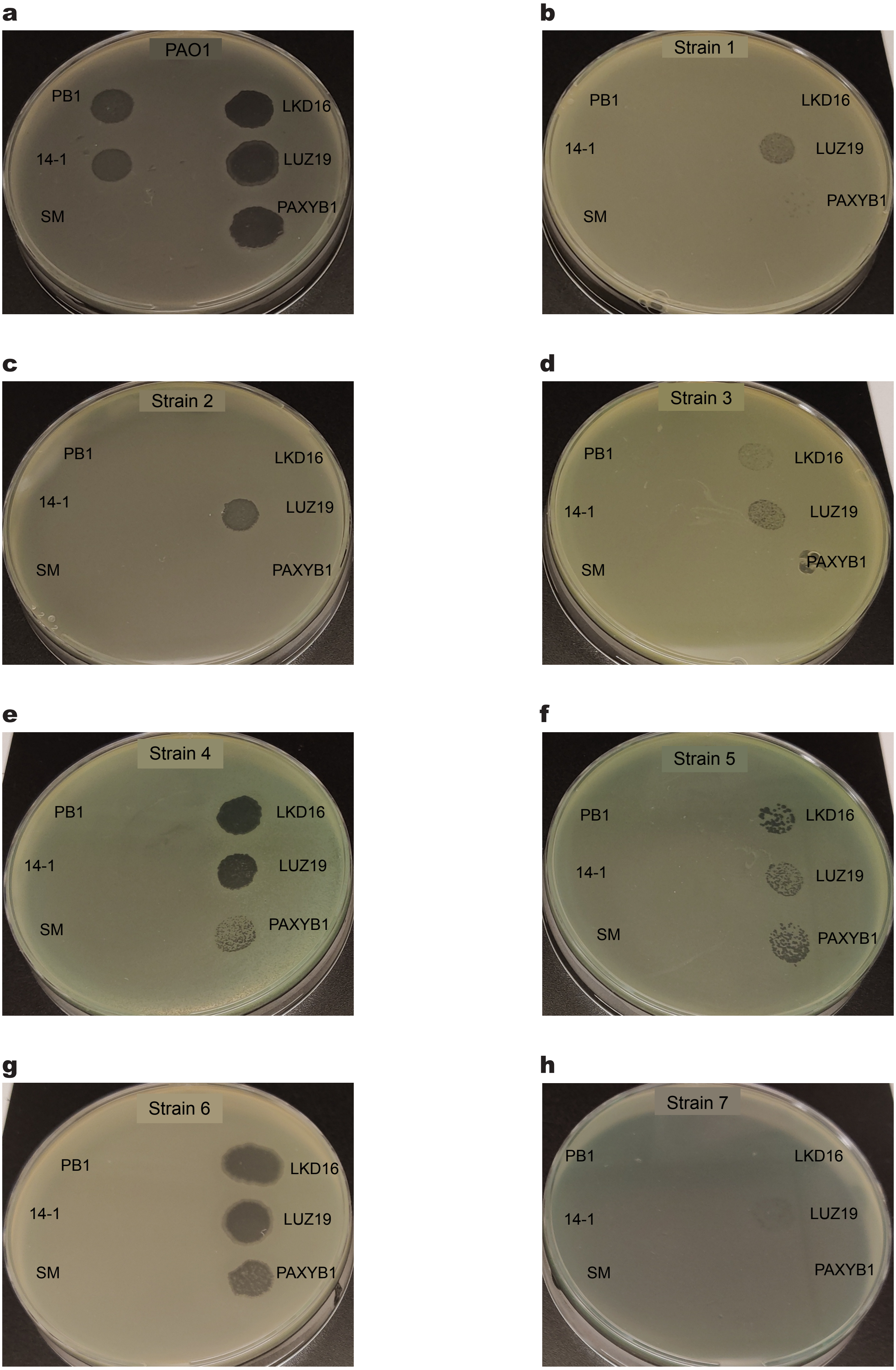
a
PAO1
PB1
LKD16
14-1
LUZ19
SM
PAXYB1
b
Strain 1
PB1
LKD16
14-1
LUZ19
SM
PAXYB1
c
Strain 2
PB1
LKD16
14-1
LUZ19
SM
PAXYB1
d
Strain 3
PB1
LKD16
14-1
LUZ19
SM
PAXYB1
e
Strain 4
PB1
LKD16
14-1
LUZ19
SM
PAXYB1
f
Strain 5
PB1
LKD16
14-1
LUZ19
SM
PAXYB1
g
Strain 6
PB1
LKD16
14-1
LUZ19
SM
PAXYB1
h
Strain 7
PB1
LKD16
14-1
LUZ19
SM
PAXYB1

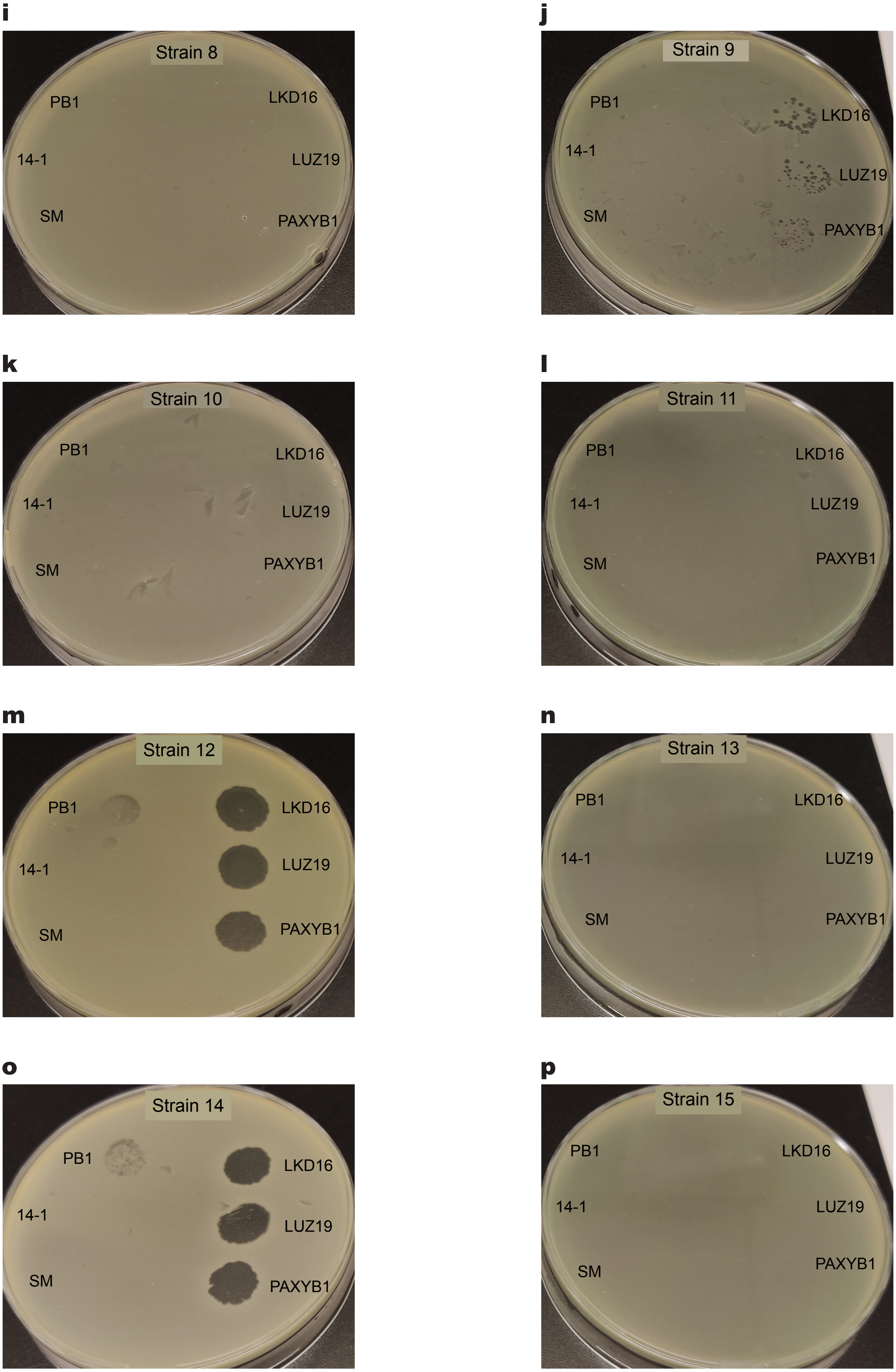
i
Strain 8
PB1
LKD16
14-1
LUZ19
SM
PAXYB1
j
Strain 9
PB1
LKD16
14-1
LUZ19
SM
PAXYB1
k
Strain 10
PB1
LKD16
14-1
LUZ19
SM
PAXYB1
l
Strain 11
PB1
LKD16
14-1
LUZ19
SM
PAXYB1
m
Strain 12
PB1
LKD16
14-1
LUZ19
SM
PAXYB1
n
Strain 13
PB1
LKD16
14-1
LUZ19
SM
PAXYB1
o
Strain 14
PB1
LKD16
14-1
LUZ19
SM
PAXYB1
p
Strain 15
PB1
LKD16
14-1
LUZ19
SM
PAXYB1

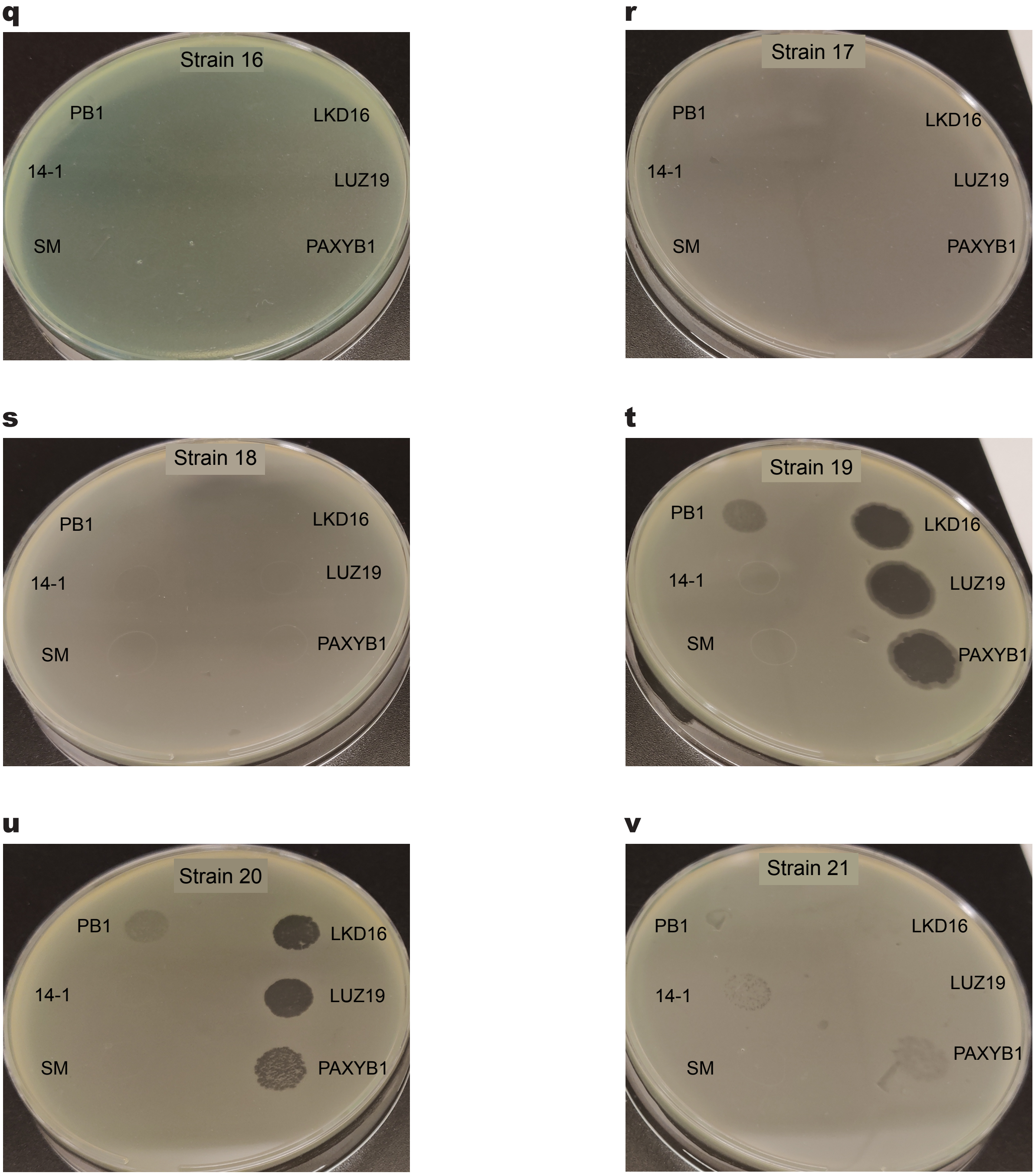

Figure S15: Phage susceptibility profiling of several *P. aeruginosa* clinical strains obtained from patients at the UMC Utrecht, as determined by plaque assays. a-v) Strains were inoculated into top agar and overlayed on a plate. Phages PB1, 14-1, LKD16, LUZ19 and PAXYB1 (10^6 pfu/mL, 5 μL) and SM buffer (5 μL) were spotted to detect plaque formation after overnight incubation at 37°C.

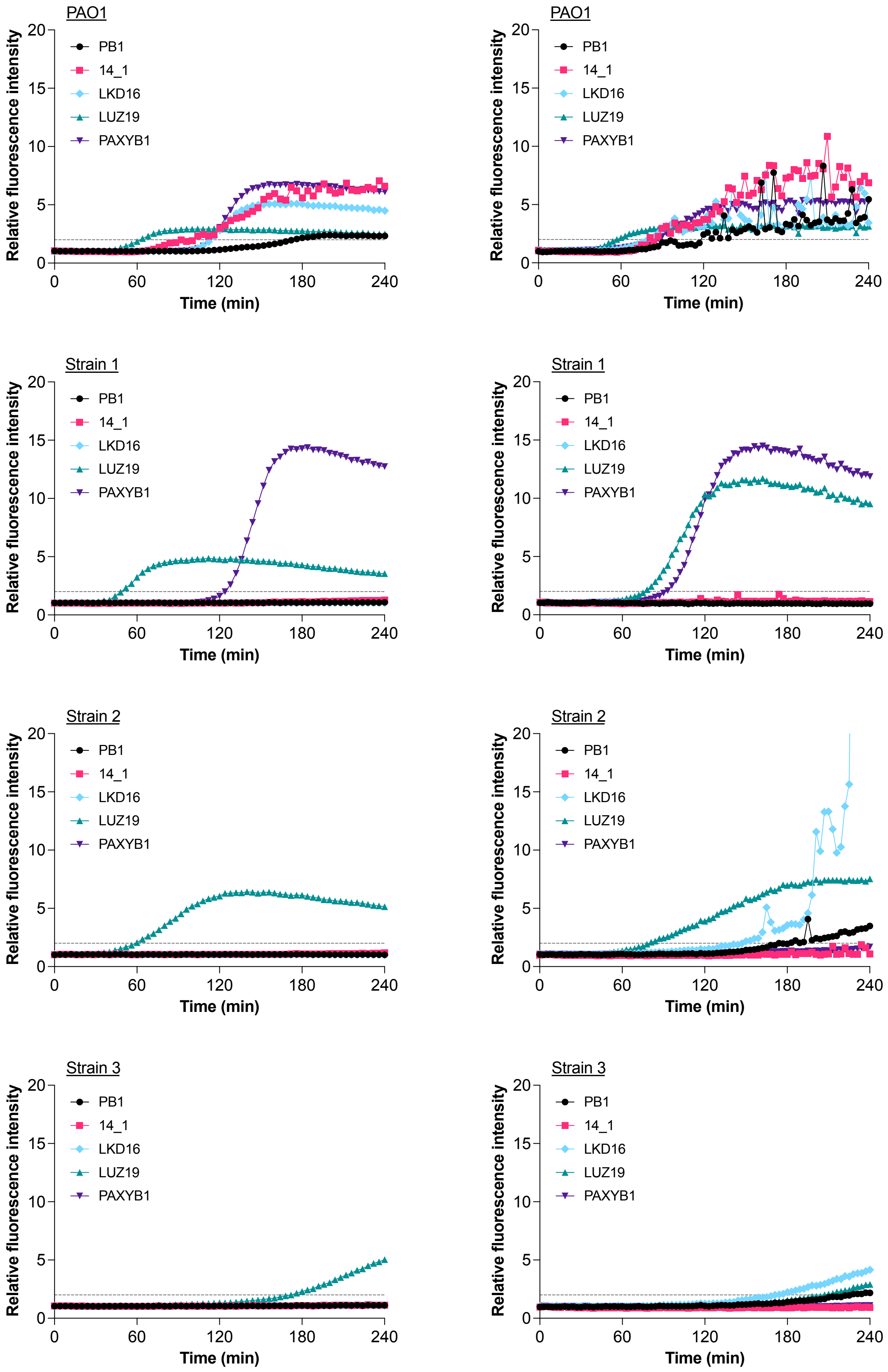
PAO1
PB1
14_1
LKD16
LUZ19
PAXYB1
Relative fluorescence intensity
0
5
10
15
20
0
60
120
180
240
Time (min)
PAO1
PB1
14_1
LKD16
LUZ19
PAXYB1
Relative fluorescence intensity
Time (min)
Strain 1
PB1
14_1
LKD16
LUZ19
PAXYB1
Relative fluorescence intensity
Time (min)
Strain 1
PB1
14_1
LKD16
LUZ19
PAXYB1
Relative fluorescence intensity
Time (min)
Strain 2
PB1
14_1
LKD16
LUZ19
PAXYB1
Relative fluorescence intensity
Time (min)
Strain 2
PB1
14_1
LKD16
LUZ19
PAXYB1
Relative fluorescence intensity
Time (min)
Strain 3
PB1
14_1
LKD16
LUZ19
PAXYB1
Relative fluorescence intensity
Time (min)
Strain 3
PB1
14_1
LKD16
LUZ19
PAXYB1
Relative fluorescence intensity
Time (min)

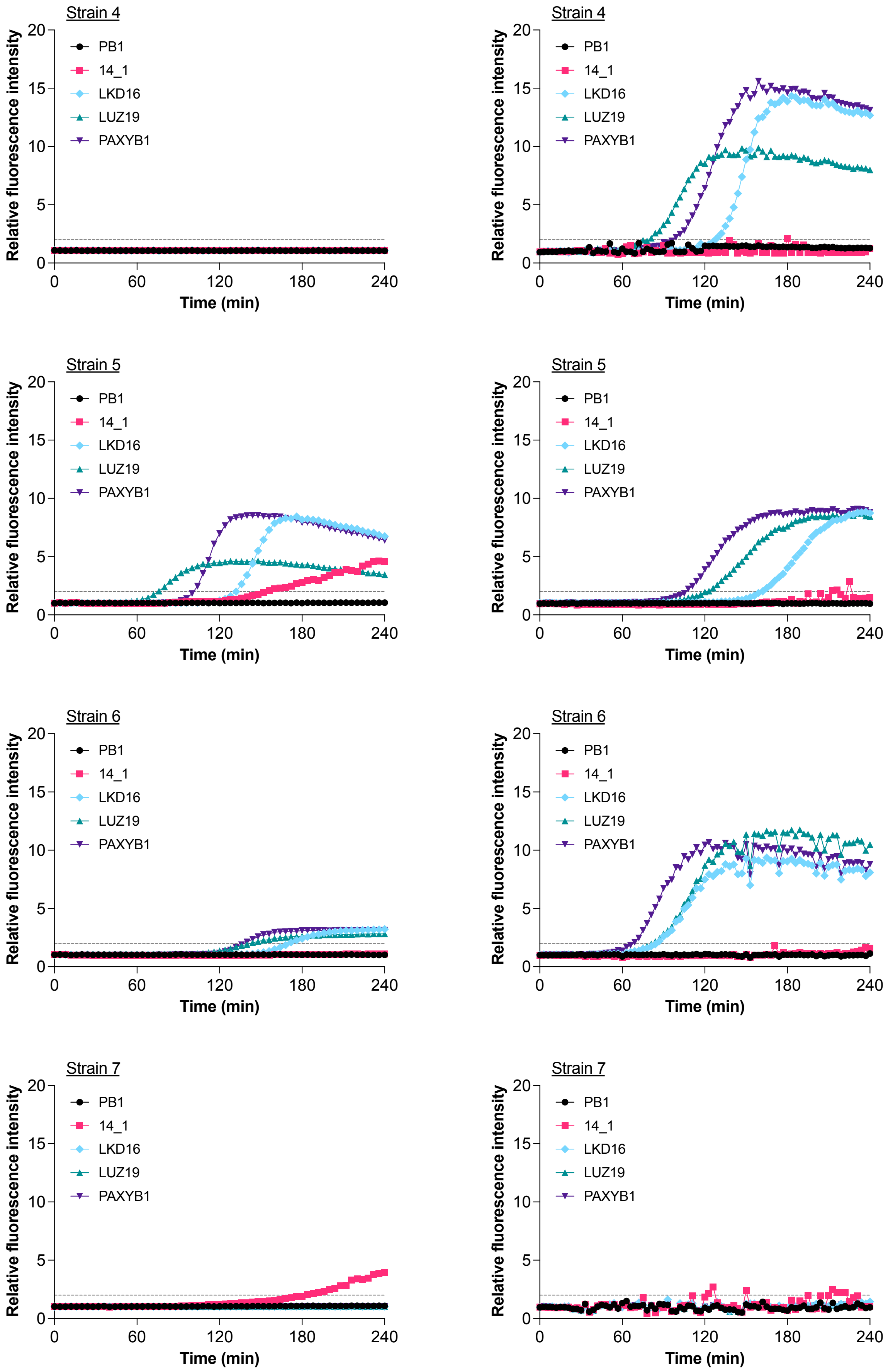
Strain 4
PB1
14_1
LKD16
LUZ19
PAXYB1
Relative fluorescence intensity
Time (min)
0
5
10
15
20
60
120
180
240
Strain 5
Strain 6
Strain 7

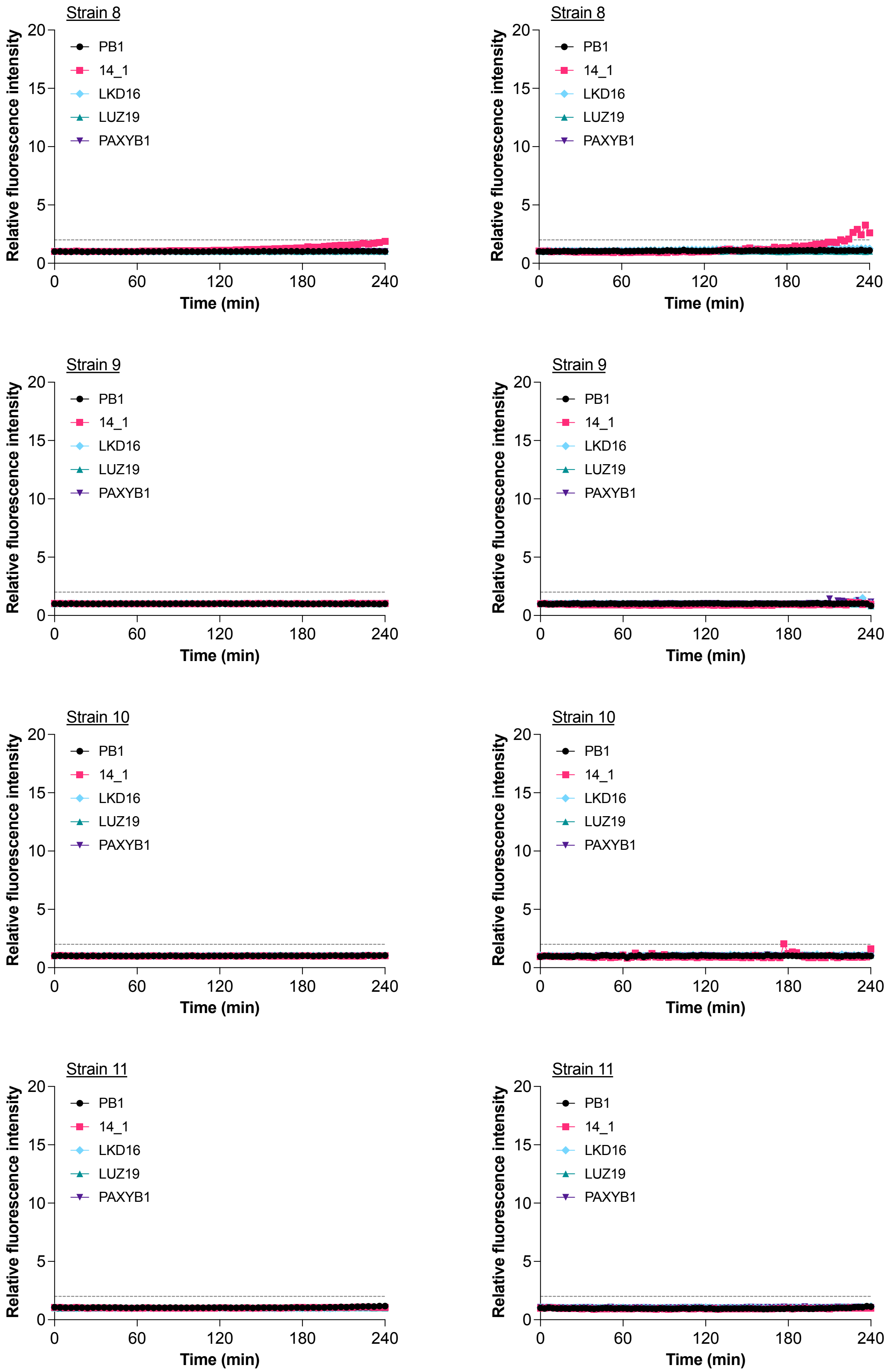

Strain 8
Strain 8
Strain 9
Strain 9
Strain 10
Strain 10
Strain 11
Strain 11
PB1
14_1
LKD16
LUZ19
PAXYB1
Relative fluorescence intensity
Time (min)
0
5
10
15
20
0
60
120
180
240

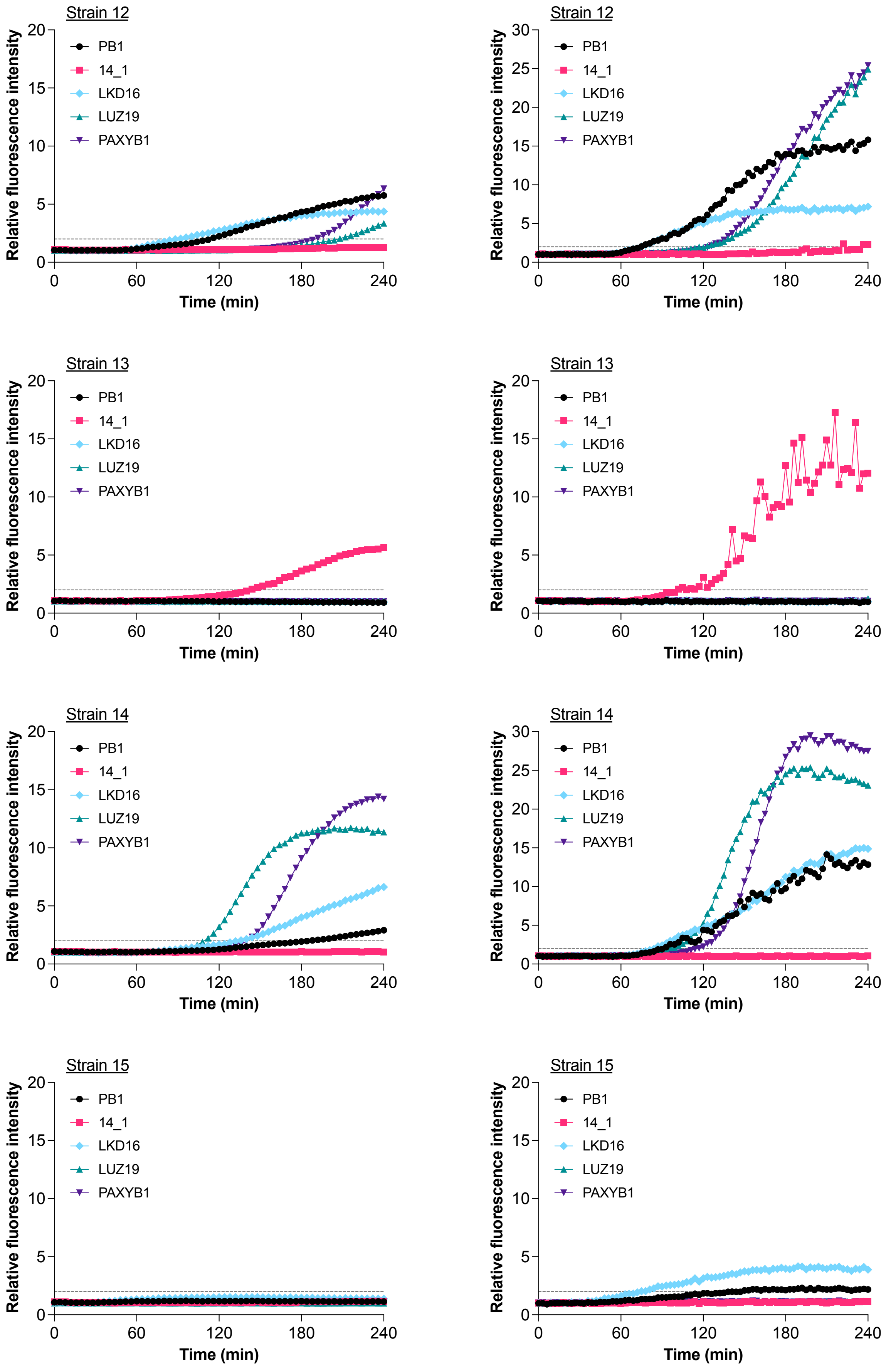
Strain 12
Relative fluorescence intensity
Time (min)
PB1
14_1
LKD16
LUZ19
PAXYB1
Strain 12
Relative fluorescence intensity
Time (min)
PB1
14_1
LKD16
LUZ19
PAXYB1
Strain 13
Relative fluorescence intensity
Time (min)
PB1
14_1
LKD16
LUZ19
PAXYB1
Strain 13
Relative fluorescence intensity
Time (min)
PB1
14_1
LKD16
LUZ19
PAXYB1
Strain 14
Relative fluorescence intensity
Time (min)
PB1
14_1
LKD16
LUZ19
PAXYB1
Strain 14
Relative fluorescence intensity
Time (min)
PB1
14_1
LKD16
LUZ19
PAXYB1
Strain 15
Relative fluorescence intensity
Time (min)
PB1
14_1
LKD16
LUZ19
PAXYB1
Strain 15
Relative fluorescence intensity
Time (min)
PB1
14_1
LKD16
LUZ19
PAXYB1

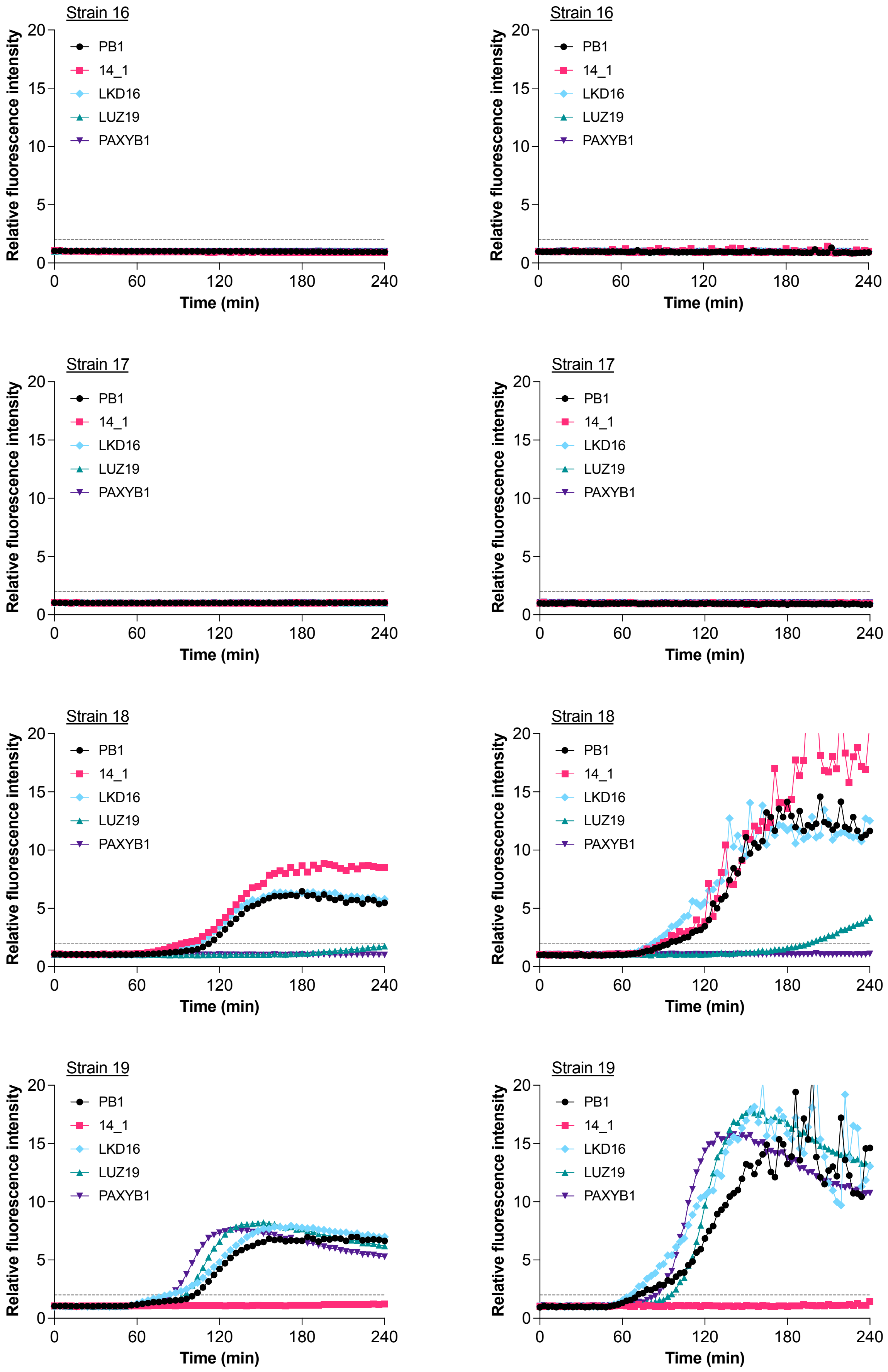
Strain 16
PB1
14_1
LKD16
LUZ19
PAXYB1
Relative fluorescence intensity
Time (min)
Strain 16
PB1
14_1
LKD16
LUZ19
PAXYB1
Relative fluorescence intensity
Time (min)
Strain 17
PB1
14_1
LKD16
LUZ19
PAXYB1
Relative fluorescence intensity
Time (min)
Strain 17
PB1
14_1
LKD16
LUZ19
PAXYB1
Relative fluorescence intensity
Time (min)
Strain 18
PB1
14_1
LKD16
LUZ19
PAXYB1
Relative fluorescence intensity
Time (min)
Strain 18
PB1
14_1
LKD16
LUZ19
PAXYB1
Relative fluorescence intensity
Time (min)
Strain 19
PB1
14_1
LKD16
LUZ19
PAXYB1
Relative fluorescence intensity
Time (min)
Strain 19
PB1
14_1
LKD16
LUZ19
PAXYB1
Relative fluorescence intensity
Time (min)

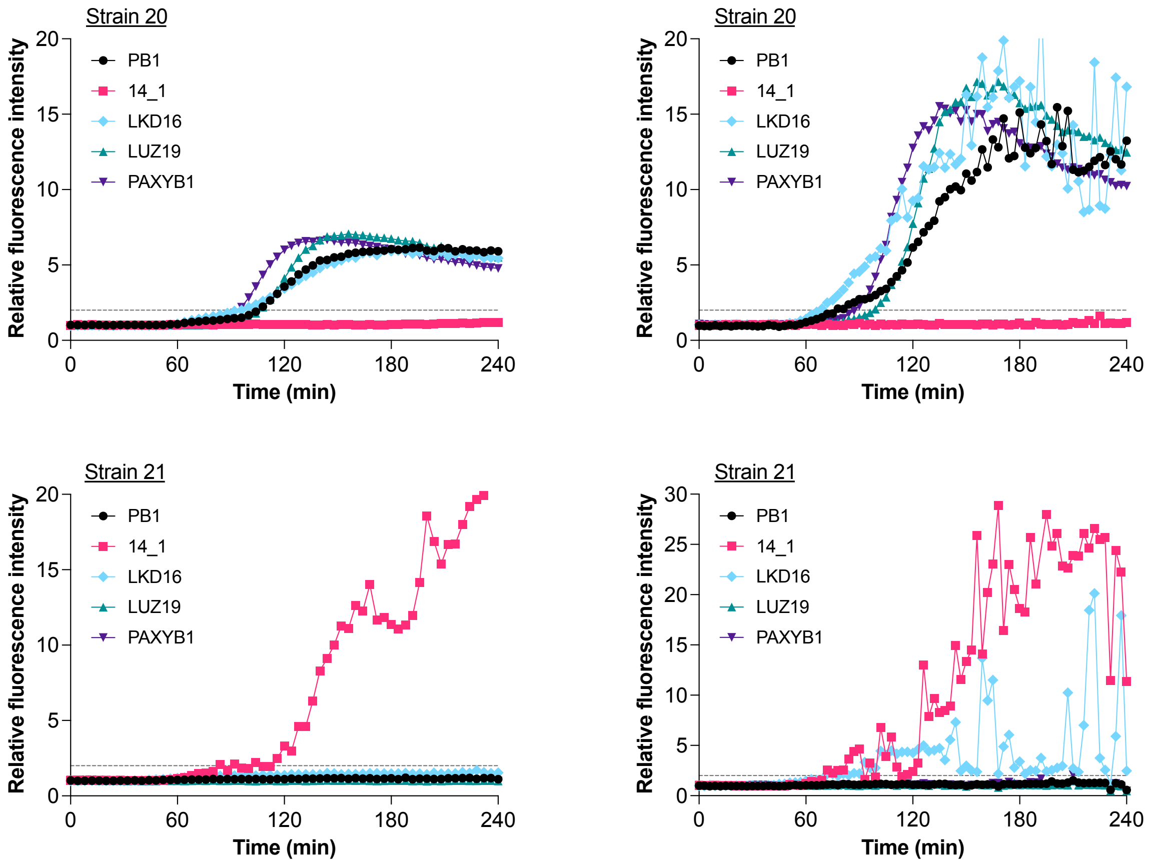

Figure S16: Phage susceptibility profiling of several *P. aeruginosa* clinical strains obtained from patients at the UMC Utrecht, as determined by fluorescent DNA dye assay. Strains were incubated with phages PB1, 14-1, LKD16, LUZ19 or PAXYB1 at an MOI of 1 at 37°C in the presence of Sytox green. Fluorescence intensity was measured using a fluorometer, and values were divided by control signal to obtain relative fluorescence intensity. Black dotted line represents threshold for phage-mediated damage (relative fluorescence = 2). A representative graph of at least three independent experiments is shown. Two additional replicates to figure S14 are shown.
